# Comparisons between retinal vessel calibers and various optic disc morphologic parameters with different optic disc appearances: The Glaucoma Stereo Analysis Study

**DOI:** 10.1371/journal.pone.0250245

**Published:** 2021-07-29

**Authors:** Kazunobu Sugihara, Yasuyuki Takai, Ryo Kawasaki, Koji Nitta, Maki Katai, Yasushi Kitaoka, Yu Yokoyama, Kazuko Omodaka, Tomoko Naito, Takehiro Yamashita, Shiro Mizoue, Aiko Iwase, Toru Nakazawa, Masaki Tanito

**Affiliations:** 1 Department of Ophthalmology, Shimane University Faculty of Medicine, Izumo, Japan; 2 Department of Vision Informatics, Osaka University Graduate School of Medicine, Osaka Japan; 3 Department of Ophthalmology, Fukui-ken Saiseikai Hospital, Fukui, Japan; 4 Department of Ophthalmology, NTT Medical Center Sapporo, Sapporo, Japan; 5 Department of Ophthalmology, St. Marianna University School of Medicine, Kawasaki, Japan; 6 Department of Ophthalmology, Tohoku University Graduate School of Medicine, Sendai, Japan; 7 Grace Eye Clinic, Okayama, Japan; 8 Department of Ophthalmology, Kagoshima University Graduate School of Medical and Dental Sciences, Kagoshima, Japan; 9 Department of Ophthalmology, Ehime University Graduate School of Medicine, Toon, Japan; 10 Tajimi Iwase Eye Clinic, Gifu, Japan; Massachusetts Eye & Ear Infirmary, Harvard Medical School, UNITED STATES

## Abstract

The Glaucoma Stereo Analysis Study (GSAS) is a multicenter collaborative study of the characteristics of glaucomatous optic disc morphology using a stereo fundus camera. This study evaluated the retinal vessel calibers and correlations using GSAS fundus photographs between retinal vessels and 38 optic nerve head (ONH) morphologic parameters comprehensively. In all 240 eyes, the mean central retinal arteriolar equivalent (CRAE) and central retinal venular equivalent (CRVE) were 138.4 and 216.5 μm, respectively; the CRAE correlated with age, visual field scores and 19 ONH parameters and CRVE correlated with age, intraocular pressure, visual field scores and 11 ONH parameters. Among the different optic disc appearances including focal ischemia (FI) (n = 53, 22%), generalized enlargement (GE) (n = 53, 22%), myopic glaucoma (MY) (n = 112, 47%), and senile sclerosis (SS) (n = 22, 9%), the CRAE did not differ significantly; CRVE was significantly narrower in SS than in FI and MY. In FI, GE, MY, and SS disc types, CRAE correlated with 3, 14, 9, and 2 ONH parameters, respectively, and CRVE corelated with 9, 0, 12, and 6 ONH parameters, respectively. We confirmed previous observations on the effect of retinal vessel narrowing on glaucomatous changes in the ONH and visual field. The associations between retinal vessel caliber and ONH morphologic parameters vary among different optic disc appearances, suggesting different effects of vascular changes in each disc type.

## Introduction

Glaucoma is a leading cause of irreversible blindness in Japan and worldwide [1, 2]. Optic disc damage due to retinal ganglion cell (RGC) and RGC axon loss is characteristic of glaucoma [3], making it essential to detect glaucomatous optic neuropathy (GON) using different modalities for disease diagnosis. GON accompanies alterations in the optic disc morphologic parameters that measure depth, i.e., excavation of optic disc cupping; however, performing ophthalmoscopy or subjectively examining one fundus photograph monoscopically may underestimate optic disc cupping and glaucoma severity [4, 5]. Using a noninvasive noncontact imaging technique to topographically analyze optic discs that does not require dilation of the pupils, such as simultaneous stereo fundus camera, provides excellent reproducibility and interexaminer consistency [6], making it promising to objectively assess the morphologic changes in GON. This technique was used in a multicenter collaborative study, the Glaucoma Stereo Analysis Study (GSAS), to assess various optic disc morphologic parameters in Japanese patients with primary open-angle glaucoma (POAG) [7–11].

There are substantial differences among patients with glaucoma in the patterns and progression of visual field defects and prevalence of risk factors, such as the intraocular pressure (IOP) and refractive error [12, 13]. A previous study suggested classifying various GONs as follows into subgroups based on the appearance of the optic disc: focal ischemic (FI), generalized enlargement (GE), myopic glaucoma (MY), and senile sclerosis (SS) [14]. Eyes of patients with differences in the appearance of the optic disc, selected based on only the assessment of fundus photographs, exhibited different demographic characteristics, prevalence rates of some systemic and ocular risk factors, IOP levels, and patterns of visual field damage [14–21]. Therefore, classifying GONs based on the appearance of the optic disc may facilitate more accurate diagnoses and better disease management because it may reflect different pathologic mechanisms of glaucoma.

It has been suggested that vascular pathologies or blood flow changes may play roles in glaucoma development and progression. In normal-tension glaucoma (NTG), optic disc hemorrhage [22], migraine [23], high frequency of nighttime hypotension [24], lower recovery rate from a cold recovery test [25], lower blood flow velocities, and higher resistive indices in patients with progressive visual field defects [26] may be associated with the pathology. Reduced retrobulbar and retinal blood flow over time can be associated with structural glaucomatous progression [27]; oral calcium channel blockers were reported to maintain the optic disc rim, visual fields, and posterior choroidal circulation in OAG [28]. Jonas et al. reported peripapillary retinal vessel narrowing in glaucomatous eyes [29, 30]. Analyses of optic disc photographs showed that arteriolar narrowing was more common in patients with glaucoma than ocular hypertension [31]. Large-scale population-based studies have reported associations between retinal arteriolar and/or venular narrowing and elevated glaucoma prevalence [32–35] and incidence [36].

Vessel caliber measurements can be obtained using fundus photography [29, 30], retinal vessel analyzer [37], retinal oximetry [38, 39], scanning laser ophthalmoscopy [40], spectral-domain optical coherence tomography (OCT) [41, 42], OCT angiography [43], Doppler OCT [43], and adaptive optics imaging [44]. Analysis of digital color fundus photographs using semi-automated methods such as interactive vessel analysis reproducibly measured arteriolar and venular calibers [45]. The current study measured retinal vessel calibers in fundus photographs obtained during the GSAS and assessed correlations between the retinal vessels and optic nerve head (ONH) morphologic parameters; the assessment was conducted in the entire dataset and for each optic disc appearance.

## Methods

### Subjects

This study adhered to the tenets of the Declaration of Helsinki. The institutional review boards of Shimane University Hospital, Fukui-ken Saiseikai Hospital, Sapporo Teishin Hospital, St. Marianna University School of Medicine, Tohoku University Graduate School of Medicine, Okayama University Graduate School of Medicine, Kagoshima University Graduate School of Medical and Dental Sciences, Ehime University Graduate School of Medicine, and Tajimi Iwase Eye Clinic reviewed and approved the research. The subjects provided written informed consent; otherwise, based on the regulations of the Japanese Guidelines for Epidemiologic Study issued by the Japanese Government, the study protocols did not require each patient provide written informed consent. The protocol was posted at the outpatient clinic to inform patients about the study. All subjects were recruited from among nine institutions (Shimane University Hospital, Fukui-ken Saiseikai Hospital, Sapporo Teishin Hospital, Hospital of St. Marianna University School of Medicine, Tohoku University Hospital, Okayama University Hospital, Kagoshima University Hospital, Ehime University Hospital, and Tajimi Iwase Eye Clinic) as reported previously [10, 11]. In this study, of the 280 GSAS enrolled subjects, 240 eyes of 240 patients with POAG were included for whom we could estimate the retinal vessel calibers. The inclusion and exclusion criteria, methods of ophthalmic examinations, diagnosis of POAG, and rules for data collection have been reported previously [7, 9]. Dataset underlying the findings described in this manuscript is shown in S1 File.

### Demographic and clinical parameters

In the current study, the following demographic and clinical parameters were extracted from the GSAS database: age, sex, spherical equivalent refractive error (SERE), treated IOP at the time of the fundus camera examination, and visual field mean deviation (MD), pattern standard deviation (PSD), and MD slope (dB/year) calculated from at least six visual fields tested during longer than 3 years. The IOP was measured using Goldmann applanation tonometry. The MD and PSD were measured using the Humphrey Visual Field Analyzer Swedish Interactive Thresholding Algorithm Standard central 30–2 or 24–2 program (Carl Zeiss Meditec, Dublin, CA, USA).

### Optic disc topography

Stereo images of the ONH were obtained using a stereo fundus camera (nonmyd WX, Kowa Company, Ltd., Aichi, Japan) that produces nonmydriatic fundus stereographs and simultaneous right and left parallactic images using one optical system to handle light paths in two directions [6]. The built-in software (VK-2 WX, prototype version, Kowa Company, Ltd.) automatically calculates the ONH morphologic parameters based on manually set contour lines for the ONH disc and cup, which in this study were determined by one of the authors (M.T.) who viewed the images stereoscopically. According to the recommendations of the Japan Glaucoma Society Guidelines for Glaucoma [46], the disc contour was delineated by the inner margin of Elschnig’s scleral ring, and the cup contour was delineated by the outer cup margin, which was indicated by the bending of the ONH vessels at the rim. The observer determined several points on the contour (typically 8–14), and the contour line then was generated automatically by software spline interpolation. Excellent intra- and inter-observer agreements between the contour delineation and the software-assisted ONH analysis were reported previously [5]. In the GSAS dataset, 38 optic disc parameters were obtained using the VK-2 WX software. The depth and volume values were calculated based on the disparity between the right and left images of the stereo image pair with a stereo matching technique, with correction for magnification by a modification of Littmann’s method using the refractive error and corneal curvature of each eye [7]. Rim decentration can range from -1, defined as rim thinning in the superotemporal area without rim thinning in the inferotemporal area, and 1, defined as rim thinning at the inferotemporal area without rim thinning at the superotemporal area; 0 indicates equally thinned rims at both the supero- and inferotemporal rims. The disc tilt angle was defined as the degree of the angle between the horizontal plane and the line drawn from the temporal to the nasal disc edge, passing through the center of the ONH; plus and minus values indicate disc tilt to the temporal and nasal sides, respectively. The details of the parameters were described previously [7]. The summary of the 38 optic disc parameters is shown in S2 File.

### Grader classification of optic disc appearance

As reported previously [8], three independent graders (T.N., K.O., and Y.Y.) classified each optic disc appearance into four different types according to the proposal of Nicolela and Drance [14], i.e., a FI disc with localized tissue loss at the superior or inferior poles and a relatively intact neuroretinal rim elsewhere; a GE disc characterized by a diffusely enlarged round cup and lack of localized defects of the neuroretinal rim; a MY disc that was tilted with temporal crescent peripapillary atrophy (PPA), excluding discs with degenerative myopia; and a SS disc with a saucerized shallow cup and diffuse neuroretinal rim tissue loss accompanied by surrounding PPA and choroidal sclerosis. Discs with features of multiple (mixed) disc types were assigned to the most prominent type. Any disagreements among the three graders were resolved by discussion. Using Cohen’s kappa statistics, the agreement among the graders was considered substantial to excellent [10, 11]. Examples of each optic disc appearance are shown in S3 File.

### Retinal vessel caliber measurement

The retinal vessel diameters were estimated on stereo fundus photographs collected during the GSAS using IVAN software developed by the Department of Ophthalmology and Visual Science, University of Wisconsin, Madison, WI, USA. Either one of the photographs from a stereo pair of 20-degree ONH photographs were used for the measurement. The detailed measurement method has been described previously [47, 48]. Briefly, all vessels 25 microns or larger were measured and passed completely through a circumferential zone 0.5 to 1 disc diameter from the optic disc margin [32]. Trained graders (K.S., Y.T.) identified each vessel as an arteriole or a venule. The six widest arteriolar and venular diameters then were defined as the central retinal arteriolar equivalent (CRAE) and the central retinal venular equivalent (CRVE), respectively, using the revised Parr-Hubbard formulas of Knudtson et al. [49]. The magnification of the optic media in an eye was corrected according to the method of Littmann, with consideration of the SERE and corneal curvature [50]. The examples of original stereophotograph (S4 File), identified vessels on IVAN software (S5 File), defined values of CRAE (S6 File) and CRVE (S7 File) by IVAN software, magnification factor calculated by Kowa nonmyd WX Tool (Kowa Company) (S8 File; in this example, the magnification factor is 1.8360 mm/300 pixel), and accompanying optic disc topography results by VK-2 WX software are found in the Supporting information files.

### Statistical analysis

All statistical analyses were performed using JMP Pro statistical software version 12.2 (SAS Institute, Inc., Cary, NC, USA). The continuous data are expressed as the means ± standard error. The continuous data were compared among the four optic disc appearances by one-way analysis of variance, which was followed by a comparison between each pair of optic disc appearances using the post-hoc Student t-test. The categorical data were compared among the four optic disc appearances using the G test. Based on Bonferroni’s method for correction of multiple comparisons, *p*<0.0083 and *p*<0.0017 were considered to be significance levels of 5% and 1%, respectively, in the post-hoc test. Correlations between the retinal vessel calibers (i.e., CRAE or CRVE) and demographic parameters (i.e., age, IOP, MD, MD slope, or PSD) or 38 optic disc parameters were assessed using the Spearman’s rank correlation coefficient; the analyses were performed in all subjects and each of the optic disc appearances.

## Results

The graders classified the 240 study eyes as follows: 53 eyes (22%) with FI, 53 eyes with GE (22%), 112 eyes with MY (47%), and 22 eyes with SS (9%). The demographic data of the subjects are summarized in Table 1. The comparisons among the different disc appearances indicated that the patient age in the MY group was significantly younger and SERE was significantly more myopic compared with the other three groups (Table 1). Other demographic parameters, including gender, visual field MD, PSD, MD slope, and IOP did not differ among the various disc appearances. The comparisons of 38 optic disc morphologic parameters among the disc types are shown in S2 File; as expected, significant differences were detected in multiple optic disc parameters.

**Table 1:** Comparison of demographic subject data among different optic disc appearances.

	Total		FI	GE	MY	SS	*p* value
N (%)	240		53 (22)	53 (22)	112 (47)	22 (9)	
Male/female	116/124		18/35	27/26	57/55	14/8	0.0720¶
Age, years	61.2 ± 9.2		62.4 ± 8.6	64.1 ± 9.4	57.8 ± 8.6	67.9 ± 6.9	<0.0001**
		*p* value‡, versus GE	0.7744	-	-	-	
		Versus MY	0.0068*	0.0001**	-	-	
		Versus SS	0.0633	0.2940	<0.0001**	-	
SERE, D	-2.8 ± 3.3		-0.7 ± 2.1	-0.7 ± 2.1	-5.0 ± 2.9	-1.9 ± 2.7	<0.0001**
		*p* value‡, versus GE	1.0000	-	-	-	
		Versus MY	<0.0001**	<0.0001**	-	-	
		Versus SS	0.2685	0.2685	<0.0001**	-	
MD, dB	-4.8 ± 3.3		-4.3 ± 3.1	-4.4 ± 3.8	-5.2 ± 3.3	-4.8 ± 3.0	0.3452†
PSD, dB	8.1 ± 4.3		8.3 ± 4.5	6.9 ± 4.5	8.7 ± 4.2	7.4 ± 3.6	0.0820†
MD slope, dB/year	-0.19 ± 0.5		-0.23 ± 0.13	-0.15 ± 0.50	-0.18 ± 0.34	-0.24 ± 0.45	0.7378†
IOP, mmHg	13.6 ± 2.5		13.6 ± 2.7	13.5 ± 2.2	13.5 ± 2.6	14.1 ± 2.5	0.7816†

The *p* values were calculated among four optic disc appearances by one-way analysis of variance (†) followed by comparison between each pair of two optic disc appearances using the post-hoc Student t-test (‡). In the post-hoc test, based on Bonferroni’s method to correct multiple comparisons, *p*<0.0083 and *p*<0.0017 were considered to be significance levels of 5% (*) and 1% (**), respectively. *P* values were calculated among the four optic disc appearances using the G-test (¶). Continuous parameters are expressed as the mean ± standard error.

FI, focal ischemic; GE, generalized enlargement; MY, myopic glaucoma; SS, senile sclerosis; SERE, spherical equivalent refractive error; MD, mean deviation; PSD, pattern standard deviation; D, diopter; dB, decibels.

The CRAE and the CRVE measured in the fundus photographs are summarized in Table 2. In all eyes, the mean CRAE and CRVE were 138.4 μm and 216.5 μm, respectively. Among the different disc appearances, the CRAE did not differ significantly; the CRVE was significantly narrower in the SS group than those in the FI and MY groups.

**Table 2:** Comparison of retinal vessel calibers among different optic disc appearances.

	Total		FI	GE	MY	SS	*p* value
CRAE, μm	138.4 ± 15.0		140.5 ± 12.1	138.5 ± 16.7	138.2 ± 15.2	134.4 ± 16.3	0.4658†
CRVE, μm	216.5 ± 21.6		219.3 ± 19.8	214.1 ± 22.0	219.3 ± 20.1	201.1 ± 25.5	0.0018†
		*p* value‡, versus GE	0.2001				
		Versus MY	0.9972	0.1367			
		Versus SS	0.0008**	0.0159	0.0003**		

The *p* values were calculated among the four types of optic disc appearances by one-way analysis of variance (†) followed by comparison between each pair of two types of optic disc appearances using the post-hoc Student t-test (‡). In the post-hoc test, based on Bonferroni’s method to correct multiple comparisons, *p*<0.0083 and *p*<0.0017 were considered to be significance levels of 5% (*) and 1% (**), respectively. Continuous parameters are expressed as the mean ± standard error.

FI, focal ischemic; GE, generalized enlargement; MY, myopic glaucoma; SS, senile sclerosis; CRAE, central retinal arteriolar equivalent; CRVE, central retinal venular equivalent.

Possible correlations between retinal vessel parameters and various demographic parameters are shown in Tables 3 (for CRAE) and 4 (for CRVE). In all eyes, the CRAE and CRVE correlated positively with each other (ρ = 0.43, *p*<0.0001); the correlation was significant for each disc appearance (ρ = 0.41–0.61) except for FI (ρ = 0.24). In all eyes, the CRAE correlated positively with the MD and MD slope and negatively with age and PSD; the CRVE correlated positively with the IOP, MD, and MD slope and negatively with age and SERE. In men and women, the CRAE values were 138.0±15.6 μm and 138.8±14.5 μm, respectively, and the CRVE values were 217.3±22.8 μm and 215.7±20.4 μm, respectively; no gender difference was seen for the CRAE (*p* = 0.6627) and CRVE (*p* = 0.5520).

**Table 3:** Correlation between CRAE and demographic parameters in each optic disc appearance group.

	Total		FI		GE		MY		SS	
	ρ	*p* value	ρ	*p* value	ρ	*p* value	ρ	*p* value	ρ	*p* value
CRVE	0.43	<0.0001**	0.24	0.0775	0.41	0.0022**	0.46	<0.0001**	0.61	0.0028**
Age	-0.15	0.0199*	-0.18	0.2014	-0.20	0.1560	-0.10	0.2745	-0.11	0.6137
SERE	-0.04	0.5449	-0.38	0.0047**	0.09	0.5399	-0.10	0.3105	0.15	0.5124
MD	0.21	0.0013**	0.06	0.6885	0.31	0.0239*	0.16	0.0869	0.34	0.1184
MD slope	0.19	0.0030**	0.26	0.6430	0.22	0.1221	0.17	0.0668	0.07	0.7543
PSD	-0.17	0.0052**	-0.04	0.7806	-0.35	0.0102**	-0.14	0.1278	-0.17	0.4588
IOP	0.10	0.1120	0.24	0.0755	0.13	0.3443	0.03	0.7832	0.14	0.5372

The *p* values and correlation coefficients (ρ) between CRAE and each parameter were calculated using the Spearman’s rank correlation coefficient test in each disc appearance group.

* and ** indicate *p*<0.05 and *p*<0.01, respectively.

FI, focal ischemic; GE, generalized enlargement; MY, myopic glaucoma; SS, senile sclerosis; CRAE, central retinal arteriolar equivalent; CRVE, central retinal venular equivalent; SERE, spherical equivalent refractive error; MD, mean deviation; PSD, pattern standard deviation.

**Table 4:** Correlation between CRVE and demographic parameters in each optic disc appearance group.

	Total		FI		GE		MY		SS	
	ρ	*p* value	ρ	*p* value	ρ	*p* value	ρ	*p* value	ρ	*p* value
CRAE	0.43	<0.0001**	0.24	0.0775	0.41	0.0022**	0.46	<0.0001**	0.61	0.0028**
Age	-0.31	<0.0001**	-0.36	0.0072**	-0.19	0.1759	-0.26	0.0054**	-0.37	0.0876
SERE	-0.18	<0.0001**	-0.26	0.0630	-0.15	0.2778	-0.20	0.0358*	-0.34	0.1245
MD	0.15	0.0164*	0.18	0.2080	0.18	0.2024	0.17	0.0783	0.21	0.3375
MD slope	0.19	0.0038**	0.22	0.1217	0.30	0.0305*	0.15	0.1192	0.11	0.6355
PSD	-0.10	0.1287	-0.15	0.2933	-0.15	0.2744	-0.17	0.0737	0.08	0.7227
IOP	0.18	0.0050**	0.18	0.1902	0.36	0.0076**	0.21	0.0295*	-0.13	0.5671

The *p* values and correlation coefficients (ρ) between CRVE and each parameter were calculated using the Spearman’s rank correlation coefficient test in each disc appearance group.

* and ** indicate *p*<0.05 and *p*<0.01, respectively.

FI, focal ischemic; GE, generalized enlargement; MY, myopic glaucoma; SS, senile sclerosis; CRAE, central retinal arteriolar equivalent; CRVE, central retinal venular equivalent; SERE, spherical equivalent refractive error; MD, mean deviation; PSD, pattern standard deviation.

Possible correlations between retinal vessel parameters and various optic disc morphologic parameters are shown in S10 (for CRAE) and S11 (for CRVE) Files. The parameters that correlated significantly with CRAE and CRVE were summarized in Tables 5 and 6, respectively. In all eyes, the CRAE correlated significantly with 19 ONH parameters (Table 5), and CRVE correlated significantly with 11 ONH parameters (Table 6). In each optic disc type, multiple parameters correlated with the CRAE and/or CRVE with various patterns. The numbers of disc morphologic parameters that correlated significantly with CRAE or CRVE in each disc appearance group are shown in Table 7. In the GE group, the CRAE correlated with 14 optic disc parameters while CRVE didn’t correlate with any parameters. In contrast, more optic disc parameters correlated with the CRVE than the CRAE in the three other disc types.

**Table 5:** Parameters significantly correlated with CRAE (sorted by effect size).

Total		FI		GE		MY		SS	
Positive correlation									
Rim area	ρ = 0.33			Minimum rim-disc ratio angle	ρ = 0.40	Rim area	ρ = 0.29	Superior minimum rim-disc ratio	ρ = 0.54
Minimum rim-disc ratio angle	0.24			Rim area	0.39	Superior minimum rim-disc ratio	0.24	Rim volume	0.46
Superior rim width	0.21			Rim-disc area ratio	0.35	Minimum rim-disc ratio angle	0.23		
Minimum rim-disc ratio	0.21			Inferior rim width	0.34	Disc volume	0.23		
Rim-disc area ratio	0.21			Minimum rim-disc ratio	0.33	Disc area	0.22		
Superior minimum rim-disc ratio	0.20			inferior minimum rim-disc ratio	0.30	Superior minimum rim-disc ratio angle	0.20		
Rim-disc ratio of section 2	0.20			Rim volume	0.30	Superior rim width	0.20		
Rim volume	0.19			Rim-disc ratio of section 5	0.29	Rim-disc ratio of section 2	0.20		
inferior minimum rim-disc ratio	0.15			Rim-disc ratio of section 4	0.28	Rim volume	0.20		
Disc volume	0.15								
Rim-disc ratio of section 6	0.15								
Rim-disc ratio of section 1	0.13								
Disc area	0.13								
Vertical disc width	0.13								
Negative correlation									
Rim category	ρ = -0.25	Rim category	ρ = -0.33	Horizontal cup-disc ratio	ρ = -0.37				
DDLS stage	-0.25	DDLS stage	-0.33	Rim category	-0.37				
Cup-disc area ratio	-0.21	Rim decentering (absolute value)	-0.29	DDLS stage	-0.37				
Vertical cup-disc ratio	-0.19			Cup-disc area ratio	-0.36				
Horizontal cup-disc ratio	-0.18			Vertical cup-disc ratio	-0.30				

The parameters significantly (*p*<0.05) correlated with CRAE by the Spearman’s rank correlation coefficient test are shown. The parameters are sorted by the effect size [i.e., correlation coefficients (ρ)].

FI, focal ischemic; GE, generalized enlargement; MY, myopic glaucoma; SS, senile sclerosis; CRAE, central retinal arteriolar equivalent; CRVE, central retinal venular equivalent; DDLS, disc damage likelihood scale.

**Table 6:** Parameters significantly correlated with CRVE (sorted by effect size).

Total		FI		GE		MY		SS	
Positive correlation									
Rim area	ρ = 0.31	Rim area	ρ = 0.37			Disc area	ρ = 0.37	Rim volume	ρ = 0.49
Rim volume	0.25	Depthmap maximum	0.34			Horizontal disc width	0.35	Disc area	0.48
Disc volume	0.25	Depthmap minimum	0.34			Rim area	0.33	Superior minimum rim-disc ratio	0.46
Depthmap maximum	0.21	Rim-disc ratio of section 4	0.34			Vertical disc width	0.32	Horizontal disc width	0.46
Depthmap minimum	0.21	Rim volume	0.31			Disc volume	0.30	Rim area	0.44
Vertical disc width	0.20	Disc volume	0.29			Cup volume	0.28	Vertical disc width	0.42
Disc area	0.19	Rim-disc area ratio	0.28			Cup area	0.25		
Horizontal disc width	0.14					Maximum cup depth	0.25		
Rim-disc area ratio	0.13					Superior minimum rim-disc ratio angle	0.24		
						Depthmap maximum	0.24		
						Depthmap minimum	0.24		
						Mean cup depth	0.21		
Negative correlation									
Horizontal cup-disc ratio	ρ = -0.13	Horizontal cup-disc ratio	ρ = -0.30						
Cup-disc area ratio	-0.13	Cup-disc area ratio	-0.28						

The parameters significantly (*p*<0.05) correlated with CRVE by the Spearman’s rank correlation coefficient test are shown. The parameters are sorted by the effect size [i.e., correlation coefficients (ρ)].

FI, focal ischemic; GE, generalized enlargement; MY, myopic glaucoma; SS, senile sclerosis; CRVE, central retinal venular equivalent; CRAE, central retinal arteriolar equivalent; DDLS, disc damage likelihood scale.

**Table 7:** Number of optic disc parameters significantly correlated with the CRAE or CRVE.

	FI	GE	MY	SS
CRAE	3	14	9	2
CRVE	9	0	12	6

## Discussion

In all eyes, a moderate (ρ = 0.43) but significant correlation was seen between the CRAE and CRVE; surprisingly few studies have reported this correlation previously. Narrowing of the CRAE and CRVE was associated with a larger cup-to-disc area ratio and smaller rim-to-disc area ratio or smaller rim volume. An association between narrower retinal vessels and glaucoma was seen in the arterioles and venules of subjects with glaucoma [34], OAG [32], POAG [30], or NTG [51]; and in the arterioles of subjects with glaucoma [33], POAG [38], NTG [38, 42, 52], or primary angle-closure glaucoma [38]. Regarding the morphologic disc parameters, an association between narrower retinal vessels and larger cup-to-disc ratio was found in the arterioles and venules of subjects with glaucoma [34], and in narrower arterioles and larger cup/smaller rim areas in POAG [29]. Other studies have reported an association between narrower retinal vessels and thinner retinal nerve fiber layer (RNFL) thickness in both the arterioles and venules of general populations [53], non-glaucomatous eyes [54], NTG [51], and in the arterioles of subjects with NTG [52]. Collectively, the current study confirmed the previous findings regarding involvement of vascular changes in glaucoma.

The current study showed that narrower CRAE and CRVE were associated with steeper MD slopes, and narrower CRVE were associated with smaller disc areas. Previously, an association between narrower retinal arterioles and worse visual fields was found in subjects with NTG [39, 52]. In subjects with asymmetric glaucoma, the retinal arteriolar diameter was narrower in eyes with advanced disease [55]. We previously reported that smaller disc areas (odd ratio, 0.49/mm^2^) were associated with faster glaucoma progression in the GSAS dataset [9]. Another group reported a significant association between smaller disc diameters and narrower retinal arteriolar and venular diameters [56]. This association also was confirmed in the current study in that both the CRAE and CRVE correlated with the disc area. Although the exact underlining mechanism is uncertain, our and previous observations have suggested a possible link among ONH size, vessel vascular morphology, and visual field progression in glaucoma.

We have found significant associations between aging and narrowing of the retinal vessel diameters in both CRAE and CRVE. Age-dependent narrowing of the retinal vessels was reported previously in both the arterioles and venules in subjects with OAG [57] and non-glaucomatous subjects older than 81 years [33], and in the arterioles in NTG [52]. Using adaptive optics imaging in POAG, narrowing of the arteriolar caliber was associated with narrowing of the vascular lumen rather than thickening of the vascular wall, suggesting dysregulation of the vascular tone other than atherosclerosis was involved in the mechanism of arteriolar narrowing [44]. Decreases in the nitric oxide (NO) marker and increases in the endothelin level were found in glaucomatous eyes [58]. Disruption of the ONH microcirculation can result in ONH ischemia and ultimately development of GON [59]. Narrowing of the retinal vessels may result from dysregulation of the vascular endothelium by a disproportionate release of endogenous vasodilators such as NO and prostacyclin and/or vasoconstrictors such as endothelin [53]. A 10-year cohort study found that patients in the lowest quartile of retinal arteriolar calibers at baseline had an approximate 4-fold higher risk of developing OAG compared with those in the highest quartile of arteriolar calibers, independent of other systemic and ocular OAG risk factors [36]. This finding was explained by whether retinal vessel caliber changes preceded subtle nerve fiber layer defects (hypothesized in the vascular theory of glaucoma) or were a subsequent change secondary to the RNFL thinning [36]. Although not conclusive, we speculated that aging can associate with vessel narrowing by its effects on tone dysregulation and atherosclerosis mechanisms.

In the GE group, both CRAE and CRVE associated with visual field parameters and CRVE associated with IOP, while only CRAE associated with ONH morphologic parameters. GE tended to be associated with younger age [14] and higher baseline IOP [10]. Previous studies have found an association between narrower retinal vessels and higher IOP in the arterioles and venules of subjects with OAG [57] and NTG [51], whereas no such association was observed in glaucoma in another study [34]. In a population-based study, no significant difference in the arteriolar caliber was found between high- and low-tension glaucoma [32]. IOP-dependent changes in retinal vessels, i.e., dilation of retinal arterioles and venules with reduction of IOP, diminished in subjects 55 years and older [57]. Among the different disc appearances, we found a significant association between vessel parameter and IOP only in the GE and MY groups; thus, a heterogenous effect of IOP on each glaucoma subject and different age distribution may explain the inconsistent results in previous and current studies. A large ONH cup is the hallmark of GE; previously, normal-caliber retinal vessels were seen in eyes with non-glaucomatous large cups, while narrowing of the retinal vessels was found in glaucomatous eyes [60]. In the GE group, the ONH microcirculation was correlated with the RNFL thickness, vertical cup-to-disc ratio, and visual field MD [61]. GE is the predominant disc type among patients with severe glaucoma [62]. Thus, GE can be a good candidate to assess the IOP-dependent glaucomatous changes in the vascular blood flow, ONH/RNFL structures, and visual functions.

Rim decentration, a marker of asymmetrical rim thinning obtained by comparing the superior and inferior rim areas, associated with CRAE in FI, while no association was detected in other disc appearances. Thus, the results might detect the focal changes in the retinal vessels in this optic disc type. The correlations between the circumpapillary RNFL thickness and visual field MD were significant in the MY, GE, and SS groups, while no association was found with FI; thus, the magnitude of the structure-function relationships may differ among different disc appearances [19]. A geographic association between the location of the retinal vessel narrowing and visual field damage was found in arterioles in subjects with POAG (no association was found in the venules) [63] or NTG [52]. Focal narrowing of the retinal arterioles accompanied thinning of the ONH rim width/area in cross-sectional [29] and longitudinal studies [64]. Thus, analyses in each segmented area of the ONH, fundus photographs, and visual field may increase the chance of detecting an association between vessel caliber and other parameters in this type of optic disc.

In the MY group, the CRAE and CRVE associated with decreased rim area and/or volume. The disc damage likelihood scale in the MY group tended to be higher than the scores in the other disc types even though the visual field MD was equivalent [8]. In myopic discs, reduced ONH microcirculation measured by laser speckle flowgraphy associated significantly with losses of RNFL and visual fields in glaucoma [65, 66]. Reduced temporal rim width and RNFL thickness, the hallmarks of MY [10], were the direct risk factors of visual acuity loss in glaucoma [67]. Accordingly, this type of ONH can be a good candidate for assessing the visual acuity and retinal vessel changes in a future study.

Among the different disc appearances, the fewest optic disc morphologic parameters associated with vessel caliber parameters in the SS group, which might associate with older age of this disc group [10, 14] and age-related loss of the vascular response with IOP change [57]. Patients with SS had a higher prevalence of ischemic heart disease and systemic hypertension [14]. A population-based study reported that the major systemic determinant of narrower CRAE was higher blood pressure, while those of wider CRVE were smokers, higher blood pressure, systemic inflammation and obesity [68], suggesting the complex mechanisms of vascular diameter regulation. Using color Doppler imaging, subjects with SS had a lower diastolic velocity and a higher resistive index in their ophthalmic, central retinal, and short posterior ciliary arteries [69]; SS associated with greater circulatory abnormalities in the retrobulbar vessels with higher downstream resistance in these vessels. These circulatory abnormalities may relate to the pathogenesis of glaucoma in SS; thus, SS is potentially a good candidate for assessing a possible association between glaucoma and age-related changes in retinal vessels and systemic diseases. The fewest subjects in this disc group may have resulted in a weaker statistical power than in the other groups; therefore, the results in this study may change if more patients are included in a future study.

Ocular hypotensive medications may have vasodilatory effects [70]; most subjects in the GSAS used medication, so that the association between glaucoma and retinal arteriolar narrowing may have been underestimated in this study. Oral antihypertensive drug may increase the retinal vessel caliber [71]; some subjects in the GSAS presumably were using antihypertensive medication, although we have no data regarding systemic drug use; thus, uncontrol for systemic drug use was a study limitation. Difference in race, environmental factors, and lifestyle habits may contribute to the results [72]; all GSAS participants were Japanese; thus, the generalizability of our results must be confirmed in different datasets.

Using the GSAS dataset, we assessed the correlation between retinal vessel narrowing and topographic changes in ONH. The associations between retinal vessel calibers and optic disc morphologic parameters vary among different optic disc types, suggesting that different etiologies may underlie glaucoma in each subject.

## Supporting information

S1 File Dataset underlying the findings described in this manuscript. (PDF)

S2 File Comparison of optic nerve disc parameters among different optic disc appearances. (PDF)

S3 File Examples of each optic disc appearance. (PDF)

S4 File Examples of original stereophotograph. (JPG)

S5 File Example of identified vessels on IVAN software. (PNG)

S6 File Example of defined value of CRAE by IVAN software. (PNG)

S7 File Example of defined value of CRVE by IVAN software. (PNG)

S8 File Example of magnification factor calculation by Kowa nonmyd WX tool. (PNG)

S9 File Example of optic disc topography results by VK-2 WX software. (PNG)

S10 File Correlation between CRAE and other parameters in each optic disc appearance group. (PDF)

S11 File Correlation between CRVE and other parameters in each optic disc appearance group. (PDF)
