# Supplementary material for: Comparisons between retinal vessel calibers and various optic disc morphologic parameters with different optic disc appearances: The Glaucoma Stereo Analysis Study
Source: PLoS One. 2021 Jul 29;16(7):e0250245. doi: 10.1371/journal.pone.0250245 (PMC8320981; doi:10.1371/journal.pone.0250245)
Supplement: S1 File — (PDF) [file pone.0250245.s001.pdf]

S1 File. Dataset

CRAE	CRVE	disc type	age	sex	ref	iop	md	psd	slope	VERTICAL_DISC_WID	HORIZONTAL_DISC_WID	VERTICAL_CUP_DISC_RATIO	HORIZONTAL_CUP_DISC_RATIO	MINIMUM_RIM_DISC_RATIO	MINIMUM_RIM_DISC_RATIO_ANGLE	SUPERIOR_MINIMUM_RIM_DISC_RATIO	SUPERIOR_MINIMUM_RIM_DISC_RATIO_ANGLE	INFERIOR_MINIMUM_RIM_DISC_RATIO	INFERIOR_MINIMUM_RIM_DISC_RATIO_ANGLE	DISC_ASPECT_RATIO	CUP_ASPECT_RATIO	SUPERIOR_RIM_WID	INFERIOR_RIM_WID	CUP_AREA	DISC_AREA	RIM_AREA	CUP_DISC_AREA_RATIO	RIM_DISC_AREA_RATIO	RIM_DISC_RATIO_SECTION_0	RIM_DISC_RATIO_SECTION_1	RIM_DISC_RATIO_SECTION_2	RIM_DISC_RATIO_SECTION_3	RIM_DISC_RATIO_SECTION_4	RIM_DISC_RATIO_SECTION_5	CUP_VOLUME	DISC_VOLUME	RIM_VOLUME	MEAN_CUP_DEPTH	MAXIMUM_CUP_DEPTH	HEIGHT_VARIATION_CONTOUR	DEPTHMAP_MAX	DEPTHMAP_MIN	RIM_CATEGORY	DDLS_STAGE	RIM_DECENTERING	DISC_TILT_ANGLE	RIM_DECENTERING(abs)
83.1	154	SS	62	m	-2.4	17	-7.5	12	0.01	1.97	1.81	0.92	0.85	0.01	46	0.02	61	0.01	253	1.09	1.17	0.14	0.03	2.15	2.76	0.62	0.78	0.22	0.02	0.04	0.11	0.13	0.03	0.03	0.31	0.68	0.03	0.14	0.44	0.22	0.58	-0.4	7	5	0.19	4.73	0.19
101	231	MY	48	f	-5.3	18	-4.4	4.31	0.33	1.96	1.68	0.83	0.8	0	289	0.1	61	0	289	1.17	1.22	0.24	0.09	1.61	2.41	0.81	0.67	0.34	0.02	0.1	0.16	0.18	0.12	0.01	0.23	1.21	0.14	0.14	0.57	0.77	1.05	0.05	6	4	0.78	9.21	0.78
105	187	MY	52	m	-2.8	12	-8.5	13.5	-1	1.96	1.65	0.86	0.89	0	1	0	65	0.02	300	1.19	1.16	0.1	0.17	1.85	2.53	0.69	0.73	0.27	0.01	0.02	0.08	0.14	0.18	0.06	0.39	1.22	0.18	0.21	0.41	0.69	0.81	-0.2	8	6	-0.6	22.2	0.59
109	193	MY	61	m	-7.5	12	-4.2	7.1	-0.2	2.25	2.04	0.84	0.8	0.04	54	0.05	60	0.06	276	1.11	1.15	0.21	0.16	2	3.43	1.44	0.58	0.42	0.07	0.08	0.16	0.16	0.2	0.08	0.32	1.03	0.21	0.16	0.41	0.64	0.66	-0.3	5	3	0.02	11.8	0.02
110	208	GE	64	f	0.75	14	-5.1	11.1	-0.9	1.88	1.71	0.97	0.87	0.01	297	0.01	85	0.01	297	1.1	1.23	0.02	0.03	2.05	2.53	0.47	0.81	0.19	0.08	0.03	0.06	0.05	0.03	0.02	0.44	0.97	0.04	0.22	0.49	0.37	0.71	-0.3	6	4	0.22	9.68	0.22
110	210	MY	59	f	-3.8	13	-5	9.24	-0.5	1.82	1.61	0.78	0.73	0.04	53	0.04	61	0.12	260	1.13	1.2	0.18	0.23	1.22	2.32	1.1	0.53	0.47	0.08	0.09	0.21	0.2	0.13	0.13	0.24	0.9	0.11	0.19	0.58	0.46	0.92	-0.1	5	3	-0.2	6.51	0.22
111	190	MY	50	m	-7.3	13	-8.9	15.2	-0.1	1.67	1.3	0.92	0.81	0	0	0	60	0	299	1.28	1.45	0.03	0.11	1.15	1.66	0.52	0.69	0.31	0	0	0.13	0.21	0.2	0.03	0.13	0.54	0.09	0.12	0.28	0.46	0.59	-0.4	8	6	-0.9	14.4	0.87
111	199	GE	69	f	0.63	13	-12	11.4	-1.5	1.83	1.66	0.92	0.92	0	167	0.07	67	0	288	1.1	1.1	0.14	0.01	2.01	2.39	0.39	0.84	0.16	0.08	0.08	0.08	0.01	0.01	0.01	0.5	0.92	0.02	0.25	0.79	0.49	0.97	-0	8	6	0.78	6.97	0.78
111	203	MY	57	f	-3.6	14	-2.4	11.1	0.28	1.84	1.49	0.84	0.8	0	273	0.12	62	0	273	1.23	1.29	0.3	0	1.33	2.13	0.82	0.62	0.39	0.04	0.14	0.19	0.19	0.13	0	0.24	1.03	0.18	0.18	0.46	0.58	0.92	-0.1	7	5	0.99	15.8	0.99
112	229	GE	68	m	0	16	-2.6	4.24	-0.1	1.88	1.81	0.92	0.85	0	270	0.07	70	0	270	1.04	1.13	0.15	0	2.04	2.7	0.67	0.75	0.25	0.09	0.08	0.08	0.06	0.04	0.03	0.59	1.11	0.04	0.29	0.69	0.19	0.9	-0.1	6	4	0.46	3.78	0.46
113	189	MY	76	m	-4.5	12	-11	14.6	-0.5	1.51	1.33	0.88	0.65	0	48	0.01	60	0.01	299	1.13	1.53	0.08	0.1	0.85	1.57	0.72	0.54	0.46	0.03	0.06	0.24	0.3	0.23	0.04	0.09	0.54	0.21	0.1	0.43	0.89	0.89	-0.1	6	4	0.28	31.6	0.28
113	183	MY	67	m	-3.8	12	-2.2	4.93	0.09	1.76	1.37	0.7	0.45	0.02	60	0.02	60	0.18	284	1.28	2	0.18	0.35	0.54	1.8	1.26	0.3	0.7	0.16	0.07	0.29	0.39	0.39	0.2	0.07	0.62	0.23	0.13	0.29	0.68	0.71	-0.3	5	3	-0.5	14	0.49
113	228	MY	50	m	-9	14	-8.5	10.1	0.12	2.1	1.68	0.9	0.68	0	7	0.01	61	0.04	299	1.25	1.67	0.12	0.1	1.61	2.66	1.05	0.61	0.4	0.01	0.04	0.17	0.36	0.14	0.04	0.15	0.87	0.27	0.1	0.29	0.74	0.68	-0.3	6	4	-0	19.1	0.03
114	180	SS	72	f	-3.3	13	-3.9	5.37	-0.4	1.63	1.66	0.75	0.66	0.06	34	0.08	62	0.08	299	0.98	1.11	0.2	0.21	1.07	2.1	1.03	0.51	0.49	0.07	0.09	0.19	0.25	0.16	0.1	0.05	0.44	0.03	0.04	0.34	0.19	0.54	-0.5	5	3	-0	0.02	0.03
116	263	MY	66	m	-6.1	14	-5.1	8.9	-0.5	1.66	1.53	0.93	0.78	0	0	0	60	0	287	1.08	1.3	0.09	0.03	1.31	1.88	0.59	0.7	0.31	0	0.02	0.18	0.21	0.11	0.02	0.28	0.74	0.15	0.21	0.43	0.6	0.77	-0.2	8	6	-0.1	18.5	0.06
117	243	MY	66	f	-8.8	18	-4.4	3.74	-0.5	2.39	2.28	0.9	0.81	0	39	0	60	0.02	299	1.05	1.17	0.05	0.19	2.89	4.17	1.27	0.7	0.31	0.02	0.01	0.1	0.18	0.17	0.05	0.67	1.13	0.15	0.23	1.69	0.42	1.84	0.84	7	5	-0.8	5.4	0.78
117	164	SS	68	f	0.13	13	-2.5	6.46	-0.6	1.43	1.49	0.89	0.67	0	299	0.03	68	0	299	0.96	1.28	0.08	0.08	0.89	1.7	0.82	0.52	0.48	0.11	0.08	0.23	0.24	0.15	0.02	0.13	0.53	0.08	0.15	0.51	0.31	0.79	-0.2	7	5	0.57	2.27	0.57
118	196	GE	80	f	-0.3	12	-11	16.1	-0.1	1.65	1.49	0.9	0.89	0.01	265	0.07	63	0.01	265	1.1	1.12	0.14	0.02	1.54	1.96	0.42	0.79	0.21	0.04	0.08	0.1	0.08	0.02	0.02	0.37	0.52	0.04	0.26	0.47	0.43	0.55	-0.5	5	3	0.63	-14	0.63
118	206	GE	59	f	1	13	-2.3	6.54	-0.2	1.6	1.61	0.74	0.8	0.08	194	0.13	114	0.13	276	1	0.92	0.22	0.2	1.15	2.03	0.88	0.57	0.43	0.13	0.14	0.13	0.1	0.13	0.14	0.26	0.73	0.08	0.23	0.54	0.25	0.81	-0.2	5	3	-0	5.78	0.02
120	194	GE	61	m	-3.3	17	-8.9	10.6	-0.2	1.82	2	0.97	0.89	0	94	0	94	0.01	241	0.91	0.99	0.02	0.03	2.4	2.83	0.44	0.85	0.15	0.1	0.07	0	0.01	0.01	0.04	0.6	0.96	0.06	0.25	0.87	0.46	1.02	0.02	8	6	0.33	-9.6	0.33
120	199	MY	60	f	-5.5	12	-8.3	12	-3	1.84	1.57	0.92	0.85	0	0	0	60	0	297	1.17	1.28	0.05	0.1	1.57	2.21	0.65	0.71	0.29	0	0.06	0.15	0.16	0.13	0.02	0.5	1.32	0.23	0.32	0.54	0.91	1.02	0.02	8	6	0.48	27.4	0.48
120	191	MY	66	f	-4.9	12	-4.8	7.69	-0.5	1.73	1.65	0.79	0.73	0.02	292	0.14	62	0.02	292	1.05	1.12	0.27	0.1	1.23	2.22	0.99	0.55	0.45	0.09	0.14	0.18	0.19	0.12	0.04	0.3	0.78	0.17	0.24	0.62	0.63	0.91	-0.1	5	3	0.57	17.4	0.57
120	221	MY	56	f	-3.6	12	-4.2	10.3	-0.3	1.89	1.11	0.79	0.61	0.01	66	0.01	66	0.07	300	1.7	2.21	0.13	0.26	0.71	1.67	0.96	0.43	0.57	0.08	0.1	0.28	0.32	0.3	0.09	0.09	0.67	0.32	0.12	0.39	1.07	0.93	-0.1	5	3	0.02	39	0.02
120	209	GE	61	f	-5.3	12	-9.1	12.6	-0.2	2.06	1.92	0.97	0.84	0.01	66	0.01	66	0.01	272	1.07	1.24	0.03	0.04	2.37	3.1	0.73	0.76	0.24	0.03	0.02	0.11	0.13	0.07	0.02	0.63	0.91	0.07	0.27	0.58	0.3	0.7	-0.3	6	4	0.08	1.69	0.08
121	184	GE	71	f	-2.5	13	-2.3	3.35	0	1.85	1.75	0.93	0.96	0	197	0.03	60	0	283	1.06	1.02	0.12	0.02	2.18	2.52	0.34	0.86	0.14	0.02	0.04	0.06	0.04	0.05	0.01	0.48	0.95	0.05	0.22	0.46	0.57	0.67	-0.3	7	5	0.73	12.3	0.73
121	181	MY	54	m	-4.3	14	-12	10.3	-0.1	2.03	1.67	0.9	0.88	0.01	285	0.04	64	0.01	285	1.22	1.25	0.15	0.05	2.09	2.72	0.63	0.77	0.23	0.02	0.05	0.08	0.1	0.11	0.02	0.68	1.18	0.08	0.32	0.76	0.41	0.98	-0	6	4	0.55	7.1	0.55
121	187	MY	39	f	-6.5	15	-7.2	11.2	-0.1	1.44	1.11	0.9	0.71	0	43	0.02	60	0.01	295	1.3	1.64	0.06	0.09	0.7	1.22	0.52	0.57	0.43	0.02	0.1	0.27	0.28	0.15	0.02	0.06	0.27	0.09	0.09	0.3	0.56	0.55	-0.5	7	5	0.68	18.6	0.68
122	206	FI	64	f	2.63	12	-11	16.3	-0.8	1.58	1.37	0.79	0.75	0.03	286	0.14	60	0.03	286	1.15	1.21	0.27	0.06	1.04	1.67	0.63	0.62	0.38	0.1	0.15	0.17	0.12	0.07	0.04	0.28	0.79	0.07	0.27	0.58	0.37	0.92	-0.1	5	3	0.55	11.9	0.55
122	163	SS	77	f	0.13	11	-8.3	9.2	-0.2	1.56	1.52	0.93	0.85	0.02	78	0.02	78	0.04	294	1.03	1.13	0.05	0.06	1.45	1.88	0.43	0.77	0.23	0.03	0.02	0.06	0.12	0.08	0.04	0.29	0.41	0.02	0.2	0.45	0.38	0.53	-0.5	5	3	-0.3	-0.2	0.32
122	190	GE	70	f	0.75	11	-1.8	2.64	-0.2	1.79	1.74	0.85	0.84	0	20	0	68	0.08	300	1.03	1.05	0.04	0.23	1.67	2.46	0.79	0.68	0.32	0.01	0.01	0.11	0.17	0.19	0.11	0.4	1.27	0.21	0.24	0.45	0.73	0.9	-0.1	8	6	-0.9	10.8	0.89
123	195	MY	64	m	-3.8	15	-3.5	7.38	-0.7	1.85	1.69	0.91	0.91	0	6	0.03	60	0	289	1.1	1.1	0.14	0.03	1.99	2.4	0.41	0.83	0.17	0	0.05	0.11	0.09	0.02	0.01	0.41	0.45	0.02	0.29	0.51	0.48	0.5	-0.5	8	6	0.71	-14	0.71
123	217	FI	68	f	-0.5	12	-3.6	9.12	-0	1.8	1.48	0.81	0.78	0	298	0.15	60	0	298	1.21	1.26	0.28	0.06	1.23	2.06	0.83	0.6	0.4	0.08	0.15	0.17	0.19	0.08	0.01	0.23	0.92	0.1	0.19	0.5	0.32	0.89	-0.1	6	4	0.87	10.4	0.87
123	208	FI	64	f	0.5	11	-0.8	4.45	-0.2	2.04	2.13	0.82	0.77	0.01	295	0.14	104	0.01	295	0.96	1.02	0.29	0.09	1.92	3.45	1.53	0.56	0.44	0.12	0.15	0.14	0.16	0.16	0.04	0.69	1.62	0.25	0.36	0.73	0.47	1.06	0.06	6	4	0.59	7.19	0.59
123	197	GE	67	f	2.38	13	0	1.79	0.01	1.97	2.07	0.87	0.79	0	243	0.11	60	0	243	0.95	1.05	0.25	0.01	2.25	3.17	0.93	0.71	0.29	0.09	0.12	0.12	0.1	0.01	0.02	0.68	0.99	0.07	0.3	0.73	0.47	0.85	-0.1	7	5	0.66	-6.4	0.66
123	193	MY	70	m	-6.8	16	-3.7	6.6	-0.2	1.84	1.3	0.66	0.77	0.02	324	0.16	60	0.04	300	1.42	1.22	0.38	0.24	0.94	1.89	0.94	0.5	0.5	0.06	0.18	0.2	0.18	0.21	0.08	0.17	0.61	0.23	0.18	0.57	0.88	0.95	-0	5	3	0.38	29.8	0.38
123	203	MY	67	m	-1.1	14	-3.3	6.62	-0.1	1.62	1.11	0.7	0.69	0	0	0.13	60	0.03	288	1.46	1.49	0.34	0.15	0.63	1.39	0.76	0.45	0.55	0.02	0.16	0.29	0.31	0.26	0.06	0.09	0.61	0.24	0.15	0.36	0.68	0.91	-0.1	7	5	0.51	26.6	0.51
123	194	GE	77	m	-0.3	11	-12	8.28	-0.5	1.68	1.78	0.84	0.76	0	271	0.15	105	0	271	0.94	1.05	0.26	0.01	1.48	2.35	0.87	0.63	0.37	0.08	0.16	0.15	0.16	0.06	0.01	0.53	1.12	0.14	0.36	0.87	0.41	1.19	0.19	6	4	0.83	7.87	0.83
124	212	FI	56	m	-1.3	17	-4.4	7.77	-0.4	1.99	1.89	0.88	0.77	0	272	0.11	111	0	272	1.05	1.19	0.24	0.01	1.92	2.95	1.04	0.65	0.35	0.09	0.12	0.12	0.14	0.07	0.03	0.31	0.63	0.08	0.16	0.43	0.26	0.56	-0.4	6	4	0.6	-0.9	0.6
124	171	GE	75	m	0.75	9	-2.1	5.88	-0	1.77	1.78	0.83	0.74	0.04	64	0.04	64	0.07	298	1	1.12	0.15	0.15	1.42	2.42	1	0.59	0.41	0.09	0.07	0.15	0.17	0.14	0.08	0.36	1.05	0.12	0.25	0.63	0.32	0.97	-0	5	3	-0.1	6.03	0.13
124	219	MY	51	f	-5	15	-6.7	12.2	-0.3	1.91	1.52	0.84	0.79	0	2	0.07	60	0	273	1.26	1.34	0.2	0.11	1.35	2.22	0.88	0.61	0.4	0	0.11	0.24	0.23	0.16	0	0.3	1.31	0.24	0.22	0.49	0.78	1.07	0.07	8	6	0.96	18.5	0.96
125	210	FI	62	m	-1.6	10	-2.9	5.54	-0.1	1.73	1.64	0.83	0.8	0	33	0	74	0.09	300	1.05	1.09	0.07	0.24	1.36	2.14	0.78	0.64	0.36	0.02	0.01	0.11	0.19	0.22	0.11	0.39	1.48	0.19	0.29	0.65	1	1.25	0.25	7	5	-0.9	13.4	0.86
125	210	MY	70	m	-4.3	13	-0.1	2.96	-0	1.63	1.64	0.86	0.81	0	260	0.09	60	0	260	0.99	1.05	0.24	0	1.33	2	0.68	0.66	0.34	0.02	0.11	0.22	0.19	0.06	0.01	0.15	0.62	0.16	0.11	0.22	0.74	0.53	-0.5	6	4	0.8	14.9	0.8
126	204	MY	60	f	-4.3	14	-12	15.7	-0.4	1.68	1.42	0.95	0.72	0	42	0	60	0.01	298	1.19	1.56	0.04	0.05	1.23	1.8	0.57	0.69	0.32	0.02	0.01	0.18	0.24	0.07	0.02	0.18	0.98	0.13	0.14	0.37	0.72	0.9	-0.1	7	5	-0.2	18.6	0.21
127	179	FI	73	f	1.75	21	-4.1	6.73	-0.4	1.52	1.6	0.82	0.84	0	283	0.14	120	0	283	0.95	0.93	0.27	0.01	1.19	1.84	0.66	0.64	0.36	0.11	0.19	0.15	0.09	0.06	0.03	0.29	0.48	0.08	0.25	0.57	0.32	0.71	-0.3	6	4	0.7	0.37	0.7
127	189	FI	70	f	0.25	11	-1.2	3.3	-0	1.77	1.63	0.88	0.83	0.01	275	0.1	64	0.01	275	1.08	1.14	0.21	0.01	1.6	2.27	0.67	0.71	0.3	0.06	0.1	0.13	0.13	0.04	0.02	0.11	0.09	0.03	0.09	0.18	0.31	0.15	-0.9	6	4	0.66	-4.5	0.66
127	227	FI	68	f	1.5	16	-3.5	3.93	-0.3	1.73	1.7	0.88	0.84	0	322	0.11	98	0	283	1.01	1.06	0.19	0.02	1.56	2.2	0.64	0.71	0.29	0.07	0.12	0.12	0.11	0.06	0.01	0.21	0.61	0.05	0.14	0.41	0.31	0.62	-0.4	7	5	0.87	6.42	0.87
127	192	GE	59	f	-5.5	16	-11	12.7	-0.3	1.89	1.64	0.92	0.78	0.01	267	0.06	76	0.01	267	1.15	1.36	0.12	0.03	1.72	2.43	0.72	0.71	0.3	0.09	0.08	0.11	0.12	0.03	0.03	0.58	1.74	0.07	0.34	0.73	0.41	1.23	0.23	5	3	0.45	10.6	0.45
127	218	GE	59	m	-0.5	15	-4.4	9.36	-0.5	1.91	1.89	0.93	0.82	0	278	0.07	85	0	278	1.01	1.14	0.14	0.01	2.02	2.84	0.83	0.71	0.29	0.07	0.07	0.1	0.12	0.07	0.03	0.58	1.09	0.06	0.29	0.65	0.27	0.85	-0.1	6	4	0.44	4.01	0.44
128	227	GE	45	m	-6.9	12	-7.2	12.2	0.04	2.32	2.02	0.94	0.9	0	0	0.03	66	0.01	263	1.15	1.2	0.11	0.04	3.05	3.73	0.68	0.82	0.18	0.01	0.04	0.08	0.09	0.05	0.02	0.83	3.02	0.11	0.27	0.7	0.67	1.34	0.34	7	5	0.5	13	0.5
128	207	MY	58	m	-1.1	11	-4.6	5.84	-0.3	1.79	1.64	0.74	0.55	0.08	294	0.12	60	0.08	294	1.09	1.49	0.31	0.15	0.92	2.27	1.34	0.41	0.59	0.1	0.15	0.27	0.34	0.22	0.08	0.12	0.41	0.1	0.13	0.48	0.32	0.65	-0.4	5	3	0.29	2.22	0.29
128	230	GE	57	f	-1.1	14	-4.3	11.3	-0.2	2.3	1.97	0.92	0.85	0	31	0	60	0.01	290	1.17	1.27	0.14	0.06	2.45	3.47	1.03	0.71	0.3	0.01	0.03	0.14	0.16	0.17	0.04	0.77	1.7	0.12	0.32	0.62	0.4	0.92	-0.1	8	6	-0.2	0.69	0.2
128	213	GE	59	f	-3.8	13	-11	15.1	-1.1	2.29	1.95	0.92	0.85	0	44	0	60	0.01	300	1.18	1.27	0.07	0.1	2.69	3.47	0.79	0.78	0.23	0.02	0.01	0.13	0.13	0.07	0.02	0.76	1.16	0.02	0.29	0.83	0.42	0.96	-0	7	5	-0.2	-6.8	0.2
128	254	MY	48	m	-1.8	13	-1.1	3.33	0.11	1.92	1.7	0.87	0.78	0.01	291	0.1	72	0.01	291	1.13	1.27	0.2	0.05	1.49	2.49	1	0.6	0.4	0.09	0.11	0.15	0.16	0.17	0.02	0.34	1.82	0.28	0.23	0.6	0.87	1.3	0.3	6	4	0.66	20.4	0.66
128	218	MY	54	f	-1.1	21	-2.4	1.55	-0.3	1.79	1.32	0.85	0.7	0.01	58	0.02	60	0.02	289	1.35	1.64	0.21	0.06	0.97	1.88	0.91	0.52	0.49	0.03	0.09	0.24	0.28	0.28	0.06	0.18	0.31	0.19	0.24	0.54	0.77	0.68	-0.3	5	3	0.13	24.1	0.13
128	227	MY	57	m	-6.4	12	-6.3	12.6	-0.9	1.59	1.17	0.71	0.67	0.01	59	0.01	60	0.14	300	1.35	1.44	0.1	0.36	0.62	1.39	0.77	0.45	0.55	0.09	0.06	0.21	0.27	0.27	0.18	0.07	0.56	0.17	0.12	0.26	0.8	0.73	-0.3	5	3	-0.5	34.9	0.46
129	193	MY	54	m	-4.3	9	-2.3	2.3	-0.1	1.52	1.5	0.7	0.51	0	32	0.08	60	0.1	300	1.01	1.4	0.19	0.26	0.59	1.73	1.14	0.34	0.66	0.07	0.14	0.36	0.4	0.34	0.15	0.06	0.3	0.27	0.11	0.27	0.68	0.54	-0.5	6	4	-0	21.8	0.05

129	202	MY	64	f	0.13	13	-1.4	7.71	0.48	1.92	1.48	0.76	0.67	0.05	63	0.05	63	0.08	298	1.3	1.47	0.18	0.29	1.04	2.23	1.19	0.47	0.53	0.08	0.09	0.2	0.25	0.26	0.13	0.22	1.16	0.24	0.21	0.58	0.54	1.11	0.11	5	3	-0.2	17.1	0.19
129	218	MY	63	m	-6.5	13	-10	13.6	-0.1	3.12	2.12	0.92	0.86	0	0	0.01	72	0.01	277	1.47	1.57	0.21	0.04	3.06	4.02	0.97	0.76	0.24	0.01	0.03	0.1	0.14	0.1	0.02	1.33	3.37	0.45	0.44	0.82	1.41	1.5	0.5	7	5	0.05	36.1	0.05
130	206	MY	52	m	-8.1	9	-4.7	11.7	-0	1.94	1.93	0.89	0.85	0.01	346	0.04	63	0.03	299	1.01	1.06	0.14	0.08	2.18	3.02	0.84	0.72	0.28	0.03	0.05	0.09	0.14	0.11	0.04	0.5	1.46	0.1	0.23	0.9	0.33	1.25	0.25	5	3	0.13	8.05	0.13
130	192	FI	66	m	0.25	11	-3.4	6.8	-0.3	1.58	1.54	0.82	0.72	0.01	285	0.13	60	0.01	285	1.03	1.17	0.25	0.03	1.04	1.87	0.83	0.55	0.45	0.12	0.14	0.18	0.17	0.13	0.04	0.17	0.74	0.11	0.16	0.48	0.44	0.85	-0.1	5	3	0.57	9.04	0.57
130	216	MY	63	m	-10	10	-4.2	8.51	-1.1	1.84	1.39	0.83	0.72	0	53	0	60	0.04	299	1.33	1.53	0.11	0.21	1.07	1.98	0.91	0.54	0.46	0.01	0.04	0.22	0.27	0.26	0.11	0.08	0.29	0.13	0.08	0.39	0.56	0.57	-0.4	7	5	-0.5	9.77	0.49
130	219	MY	61	m	-11	14	-9.7	12.7	-0	1.32	0.84	0.73	0.72	0.01	325	0.05	60	0.06	299	1.58	1.6	0.25	0.11	0.36	0.81	0.45	0.45	0.55	0.1	0.11	0.19	0.25	0.26	0.13	0.06	0.5	0.13	0.15	0.44	0.88	1.13	0.13	6	4	-0.2	44.1	0.2
130	201	FI	69	m	2.75	10	-9.9	16.4	0.09	1.81	1.5	0.8	0.65	0	252	0.18	119	0	252	1.21	1.49	0.35	0.01	1.11	2.08	0.98	0.53	0.47	0.17	0.21	0.18	0.21	0.04	0.01	0.2	0.67	0.13	0.18	0.58	0.47	0.87	-0.1	7	5	0.88	8.33	0.88
130	210	FI	58	f	0.5	10	-6.6	14.9	-0.7	1.84	1.67	0.81	0.75	0.02	275	0.15	115	0.02	275	1.1	1.19	0.31	0.03	1.4	2.44	1.04	0.58	0.43	0.13	0.17	0.15	0.12	0.11	0.06	0.28	1.03	0.14	0.2	0.41	0.38	0.78	-0.2	5	3	0.51	1.04	0.51
130	201	FI	74	f	3.5	17	-2	1.84	-0.2	1.72	1.76	0.83	0.68	0	281	0.15	100	0	281	0.98	1.21	0.28	0.01	1.36	2.37	1.01	0.57	0.43	0.11	0.16	0.16	0.19	0.08	0.01	0.34	0.75	0.17	0.26	0.64	0.62	0.87	-0.1	6	4	0.93	14.4	0.93
130	248	FI	46	f	0	13	-2.3	2.92	-0	1.62	1.43	0.75	0.62	0.01	287	0.19	105	0.01	287	1.14	1.38	0.33	0.07	0.85	1.82	0.97	0.47	0.53	0.11	0.2	0.2	0.26	0.18	0.02	0.13	0.88	0.26	0.16	0.46	0.69	1.02	0.02	6	4	0.83	16.7	0.83
131	223	FI	80	m	1	15	-9	10.2	-0.5	1.65	1.6	0.86	0.75	0.01	286	0.09	61	0.01	286	1.03	1.19	0.19	0.04	1.29	2.03	0.74	0.64	0.36	0.07	0.1	0.16	0.16	0.09	0.02	0.32	0.94	0.07	0.25	0.84	0.44	1.18	0.18	6	4	0.69	0.4	0.69
131	209	FI	66	f	-0.1	7	-7.6	13.2	0.89	1.48	1.41	0.81	0.69	0	16	0.1	60	0.02	288	1.05	1.22	0.21	0.08	0.93	1.57	0.65	0.59	0.41	0.02	0.11	0.18	0.27	0.16	0.03	0.19	0.47	0.03	0.2	0.51	0.25	0.7	-0.3	7	5	0.6	-2.7	0.6
131	212	MY	63	f	-1.8	14	-7.4	12.1	-0.5	1.9	1.29	0.87	0.79	0.01	329	0.02	60	0.02	281	1.47	1.62	0.2	0.06	1.18	1.93	0.75	0.61	0.39	0.02	0.07	0.18	0.22	0.21	0.03	0.29	0.92	0.19	0.24	0.58	0.58	1.02	0.02	6	4	0.4	17.9	0.4
131	218	MY	49	m	-3.3	14	-4.1	11	-0.1	1.86	1.53	0.88	0.81	0	15	0.03	60	0	283	1.22	1.33	0.12	0.1	1.38	2.11	0.73	0.66	0.35	0	0.07	0.21	0.21	0.14	0.01	0.2	0.54	0.11	0.15	0.53	0.87	0.76	-0.2	8	6	0.89	13.7	0.89
131	212	MY	57	m	-3	15	-4.6	12.1	-0.9	1.79	1.45	0.79	0.6	0.03	6	0.04	78	0.27	300	1.24	1.62	0.1	0.27	0.75	2.08	1.33	0.36	0.64	0.06	0.07	0.31	0.37	0.37	0.27	0.09	0.77	0.3	0.11	0.25	0.54	0.73	-0.3	5	3	-0.6	11.3	0.58
132	181	SS	71	f	-2	15	-4.7	6.34	-0.4	1.58	1.72	0.89	0.7	0	30	0.06	60	0.01	284	0.92	1.16	0.15	0.04	1.24	2.09	0.85	0.59	0.41	0.04	0.07	0.16	0.24	0.15	0.04	0.18	0.54	0.03	0.15	0.36	0.39	0.53	-0.5	6	4	0.35	-6.9	0.35
132	222	MY	54	m	-5	13	-8.7	13.7	-0.1	2.33	1.73	0.88	0.79	0.01	37	0.02	75	0.03	289	1.35	1.5	0.12	0.16	1.85	3.18	1.33	0.58	0.42	0.02	0.05	0.22	0.23	0.23	0.06	0.2	1.28	0.26	0.11	0.25	0.62	0.69	-0.3	5	3	-0.1	12.7	0.11
132	175	GE	76	m	1	11	-9	12.1	0	1.79	1.95	0.92	0.86	0	273	0.07	61	0	273	0.92	0.98	0.14	0.01	2.15	2.81	0.66	0.77	0.23	0.06	0.07	0.08	0.09	0.04	0.02	0.57	0.52	0.02	0.31	0.62	0.23	0.59	-0.4	6	4	0.63	-4.6	0.63
132	207	SS	68	m	-8.6	15	-4.7	9.16	0.11	2.05	1.94	0.89	0.8	0.02	44	0.03	63	0.04	271	1.05	1.18	0.12	0.1	2.12	3.07	0.96	0.69	0.31	0.04	0.04	0.09	0.15	0.12	0.06	0.28	0.71	0.04	0.13	0.45	0.15	0.61	-0.4	5	3	-0.3	2.31	0.31
132	210	MY	63	m	-9.4	18	-5.5	9.64	-0.8	1.83	1.35	0.85	0.8	0	12	0	60	0.02	299	1.36	1.44	0.12	0.15	1.25	1.91	0.67	0.66	0.35	0	0.02	0.15	0.2	0.2	0.08	0.27	1.07	0.21	0.21	0.54	1.03	1.13	0.13	8	6	-0.6	24	0.59
132	237	FI	64	f	1.25	14	-4.1	8.45	-0.3	1.94	1.85	0.86	0.78	0	286	0.11	112	0	286	1.05	1.16	0.24	0.03	1.75	2.84	1.09	0.62	0.38	0.13	0.13	0.12	0.14	0.11	0.01	0.37	1.09	0.12	0.22	0.49	0.55	0.78	-0.2	6	4	0.86	8.33	0.86
132	205	MY	69	m	-1.6	10	-9.4	11	-0.4	1.91	1.56	0.88	0.82	0	41	0.02	60	0.01	286	1.22	1.31	0.2	0.04	1.55	2.3	0.75	0.68	0.33	0.02	0.05	0.19	0.18	0.12	0.02	0.29	1.18	0.22	0.19	0.39	0.71	0.87	-0.1	6	4	0.44	18.6	0.44
133	240	GE	73	m	1.63	13	-12	9.55	1.61	1.78	1.66	0.85	0.85	0	277	0.12	119	0	277	1.07	1.08	0.24	0.03	1.48	2.24	0.76	0.66	0.34	0.07	0.15	0.13	0.13	0.09	0	0.29	1.07	0.11	0.2	0.47	0.37	0.86	-0.1	7	5	0.97	7.04	0.97
133	218	MY	61	m	-9	8	-9.1	13.9	-0.3	1.91	1.28	0.88	0.81	0	295	0.01	90	0	295	1.49	1.61	0.05	0.18	1.16	1.85	0.69	0.63	0.37	0.01	0.03	0.13	0.19	0.25	0.08	0.18	0.84	0.12	0.16	0.47	0.59	0.9	-0.1	6	4	-0.4	22.2	0.39
133	223	MY	59	m	-9.8	11	-2.1	5.41	0.11	1.63	1.02	0.62	0.48	0	294	0.21	60	0	294	1.6	2.06	0.36	0.26	0.36	1.15	0.79	0.31	0.69	0.12	0.23	0.31	0.36	0.29	0.07	0.02	0.21	0.12	0.07	0.12	0.55	0.36	-0.6	6	4	0.58	5.93	0.58
133	208	MY	77	f	-0.4	18	0.07	2.91	0.55	1.51	1.43	0.88	0.74	0.02	289	0.06	61	0.02	289	1.06	1.27	0.14	0.03	1.06	1.68	0.62	0.63	0.37	0.04	0.07	0.17	0.22	0.12	0.02	0.21	0.85	0.12	0.19	0.39	0.62	0.84	-0.2	5	3	0.52	17.6	0.52
133	181	SS	74	m	-1.8	12	-4.3	2.85	0.17	1.8	1.84	0.76	0.5	0.01	48	0.02	60	0.15	300	0.98	1.48	0.15	0.29	0.96	2.67	1.71	0.36	0.64	0.07	0.1	0.36	0.42	0.34	0.17	0.03	0.77	0.29	0.03	0.14	0.51	0.52	-0.5	6	4	-0.3	14.5	0.26
133	231	SS	61	m	-1.4	10	-2.7	5.94	0.09	1.87	2.01	0.88	0.77	0.01	285	0.1	60	0.01	285	0.93	1.06	0.21	0.01	2	2.99	1	0.67	0.33	0.11	0.11	0.12	0.11	0.04	0.03	0.34	0.68	0.06	0.17	0.66	0.35	0.8	-0.2	6	4	0.53	-0.7	0.53
133	242	MY	50	m	-7.3	16	-4.9	8.08	0.85	2.2	1.77	0.9	0.86	0	0	0.01	68	0.01	277	1.25	1.29	0.04	0.19	2.09	2.85	0.77	0.73	0.27	0.01	0.04	0.14	0.15	0.11	0.02	0.47	1.41	0.18	0.22	0.49	0.74	0.9	-0.1	7	5	0.49	17.9	0.49
133	243	SS	65	m	-4.8	12	-3.9	6.14	-0.2	1.84	1.53	0.91	0.71	0.01	53	0.02	62	0.02	281	1.2	1.54	0.12	0.04	1.41	2.2	0.79	0.64	0.36	0.03	0.04	0.16	0.23	0.14	0.03	0.17	0.76	0.16	0.12	0.25	0.62	0.62	-0.4	6	4	0.08	19.6	0.08
133	214	MY	62	f	-2.8	12	-5.8	10	-0.1	1.79	1.45	0.86	0.69	0.01	289	0.03	63	0.01	289	1.24	1.55	0.18	0.07	1.07	1.93	0.87	0.55	0.45	0.07	0.06	0.19	0.24	0.21	0.05	0.07	1.22	0.23	0.06	0.21	0.86	0.93	-0.1	5	3	0.07	25.5	0.07
134	242	FI	53	m	-4.3	14	-2.7	7.7	0.02	1.57	1.7	0.77	0.73	0.01	292	0.17	60	0.01	292	0.93	0.98	0.3	0.05	1.12	2.05	0.94	0.54	0.46	0.14	0.17	0.19	0.15	0.05	0.07	0.26	0.8	0.14	0.23	0.49	0.36	0.82	-0.2	5	3	0.39	4.98	0.39
134	204	MY	65	f	-5.5	16	-8.2	11.9	0.76	1.96	1.19	0.88	0.74	0	29	0	60	0.01	288	1.64	1.97	0.08	0.15	1.06	1.76	0.7	0.6	0.4	0.01	0.05	0.23	0.25	0.17	0.04	0.18	0.53	0.2	0.17	0.37	0.88	0.72	-0.3	7	5	0.2	29.1	0.2
134	227	MY	53	m	-5.3	16	-8.3	13.4	-0.4	1.75	1.66	0.78	0.73	0	284	0.14	60	0	284	1.06	1.13	0.28	0.1	1.2	2.15	0.95	0.56	0.44	0.06	0.15	0.19	0.24	0.12	0.02	0.2	0.56	0.3	0.18	0.37	0.92	0.63	-0.4	6	4	0.85	23.4	0.85
134	207	MY	50	m	-5.3	12	-3.6	4.21	-0.4	1.77	1.68	0.8	0.67	0.03	272	0.15	82	0.03	272	1.06	1.27	0.28	0.07	1.2	2.33	1.13	0.52	0.49	0.12	0.16	0.19	0.2	0.11	0.05	0.28	1.27	0.26	0.24	0.63	0.74	1.16	0.16	5	3	0.6	21.5	0.6
134	193	MY	54	m	-3.1	9	-3.5	6.59	0.17	1.78	1.14	0.69	0.67	0	10	0.1	60	0	294	1.57	1.62	0.23	0.32	0.61	1.51	0.9	0.4	0.6	0.01	0.15	0.3	0.33	0.35	0.14	0.09	0.54	0.31	0.15	0.45	0.93	0.98	-0	7	5	0.16	35.4	0.16
134	241	FI	56	f	-0.8	12	-5.4	12.1	-0.1	1.87	1.84	0.79	0.62	0.05	61	0.05	61	0.11	279	1.01	1.3	0.17	0.22	1.29	2.64	1.35	0.49	0.51	0.11	0.07	0.14	0.27	0.17	0.12	0.27	1.38	0.32	0.21	0.39	0.61	0.94	-0.1	5	3	-0.2	14.7	0.25
134	197	GE	50	m	-1.8	14	-5.3	7.76	-0	2.14	2.19	0.94	0.74	0	224	0.05	104	0	240	0.98	1.23	0.11	0.03	2.65	3.6	0.96	0.74	0.27	0.11	0.08	0.06	0.1	0	0.02	0.8	1.74	0.07	0.3	0.74	0.55	1.02	0.02	7	5	0.51	-6.2	0.51
134	191	SS	72	m	-3.5	13	-6.9	5.62	-0.3	1.68	1.51	0.94	0.75	0	18	0.01	79	0.03	267	1.11	1.4	0.04	0.06	1.37	2	0.63	0.68	0.32	0.02	0.02	0.17	0.22	0.08	0.03	0.16	0.69	0.08	0.12	0.33	0.38	0.65	-0.4	6	4	-0.2	11.2	0.2
134	198	MY	59	f	-5.3	14	-8.5	13.9	-0.1	1.83	1.82	0.93	0.85	0	28	0.03	60	0	251	1	1.09	0.13	0	1.94	2.6	0.66	0.75	0.25	0.01	0.04	0.18	0.15	0.03	0.01	0.41	0.35	0.06	0.29	0.54	0.58	0.47	-0.5	7	5	0.5	-12	0.5
135	196	MY	63	f	-5.5	14	-5.7	10.2	-0.8	1.74	1.31	0.7	0.6	0.08	278	0.18	60	0.08	278	1.33	1.54	0.35	0.18	0.67	1.67	1.01	0.4	0.6	0.12	0.19	0.26	0.28	0.19	0.1	0.08	0.29	0.12	0.11	0.29	0.41	0.48	-0.5	5	3	0.39	3.98	0.39
135	207	FI	63	f	-2.3	13	-11	15.5	-2.3	1.35	1.5	0.83	0.8	0	265	0.14	61	0	265	0.9	0.93	0.23	0.01	1.09	1.62	0.53	0.67	0.33	0.04	0.15	0.17	0.17	0.03	0	0.19	0.48	0.11	0.18	0.41	0.63	0.66	-0.3	8	6	1	7.19	1
135	251	GE	68	m	0.5	14	0.42	4.3	0.35	1.76	1.72	0.77	0.75	0.1	81	0.1	81	0.12	244	1.02	1.04	0.18	0.23	1.36	2.36	1	0.58	0.43	0.12	0.1	0.11	0.13	0.12	0.14	0.37	0.71	0.09	0.27	0.58	0.45	0.75	-0.2	5	3	-0.2	8.17	0.16
135	210	GE	63	f	-0.4	18	-2.6	6.34	-0.1	2.16	2.01	0.85	0.83	0.01	264	0.11	119	0.01	264	1.08	1.11	0.29	0.03	2.42	3.33	0.91	0.73	0.27	0.07	0.12	0.12	0.09	0.02	0.02	0.76	1.06	0.1	0.32	0.59	0.43	0.7	-0.3	5	3	0.67	0.01	0.67
135	210	MY	54	m	-6.1	13	-11	12.2	-0.2	1.75	1.16	0.77	0.75	0	0	0.15	83	0	281	1.51	1.53	0.27	0.15	0.77	1.44	0.66	0.54	0.46	0.01	0.15	0.3	0.3	0.15	0.01	0.17	0.67	0.23	0.22	0.49	0.99	1.03	0.03	8	6	0.91	37.9	0.91
135	189	SS	77	f	3	17	-10	8.39	-1.8	1.8	1.78	0.92	0.84	0	297	0.01	60	0	297	1.01	1.11	0.1	0.04	1.85	2.49	0.64	0.74	0.26	0.02	0.03	0.1	0.15	0.12	0.02	0.38	0.71	0.07	0.21	0.72	0.44	0.87	-0.1	6	4	0.23	0.49	0.23
135	208	MY	60	m	0.5	18	-6.6	8.97	0.14	2.11	1.64	0.86	0.73	0.02	282	0.09	70	0.02	282	1.29	1.52	0.21	0.08	1.61	2.66	1.04	0.61	0.39	0.1	0.1	0.12	0.16	0.14	0.05	0.57	1.87	0.16	0.35	0.76	0.39	1.31	0.31	5	3	0.29	9.04	0.29
135	207	MY	67	m	-1.3	12	0.04	3.02	0.12	1.85	1.38	0.73	0.68	0	0	0.01	61	0.09	300	1.34	1.45	0.17	0.33	0.96	1.85	0.9	0.52	0.49	0	0.06	0.2	0.31	0.23	0.15	0.13	0.54	0.2	0.14	0.45	0.77	0.79	-0.2	7	5	-0.4	22.2	0.45
135	205	MY	68	f	0.75	16	-4	13	0.04	1.81	1.57	0.78	0.72	0.05	280	0.12	62	0.05	280	1.16	1.25	0.31	0.09	1.28	2.18	0.9	0.59	0.41	0.11	0.13	0.17	0.15	0.08	0.06	0.23	1.05	0.11	0.18	0.42	0.5	0.84	-0.2	5	3	0.35	12.4	0.35
136	219	MY	65	f	-5	15	-8.5	11.7	-0.5	1.86	1.53	0.91	0.85	0	36	0	60	0.02	299	1.22	1.3	0.1	0.07	1.64	2.21	0.58	0.74	0.26	0.01	0.03	0.15	0.15	0.09	0.02	0.33	0.73	0.14	0.21	0.45	0.77	0.7	-0.3	7	5	0.16	17.1	0.16
136	235	FI	69	m	1.5	21	-9	14.2	-0.9	1.89	1.72	0.89	0.77	0	287	0.1	90	0	287	1.1	1.26	0.19	0.01	1.79	2.58	0.79	0.69	0.31	0.09	0.11	0.11	0.12	0.04	0.01	0.4	1.34	0.14	0.23	0.56	0.62	0.98	-0	6	4	0.8	7.35	0.8
136	214	MY	62	f	-3.9	14	-0.5	2.54	0.12	1.93	1.68	0.84	0.82	0.01	272	0.14	97	0.01	272	1.15	1.18	0.29	0.03	1.66	2.54	0.88	0.65	0.35	0.05	0.14	0.15	0.15	0.1	0.02	0.55	1.7	0.13	0.33	0.77	0.51	1.28	0.28	5	3	0.78	5.42	0.78
136	203	GE	75	m	-1.5	13	-5.4	8.84	-0.4	1.63	1.84	0.87	0.86	0	196	0.1	117	0.01	243	0.89	0.89	0.17	0.05	1.58	2.35	0.77	0.67	0.33	0.13	0.11	0.1	0.07	0.02	0.12	0.6	0.76	0.13	0.43	1.01	0.71	1.08	0.08	6	4	-0	-18	0.04
136	193	SS	71	m	-1.5	17	-8.6	11.6	-0.4	2.19	2.1	0.93	0.94	0.01	258	0.03	63	0.01	258	1.04	1.03	0.12	0.04	3.05	3.56	0.51	0.86	0.14	0.03	0.04	0.06	0.04	0.02	0.04	0.72	0.67	0.04	0.28	0.57	0.55	0.55	-0.5	6	4	-0	-13	0.03
137	229	GE	65	m	-0.8	13	-0.5	1.54	-0.2	2.06	2.01	0.78	0.8	0.06	34	0.08	60	0.11	269	1.03	1	0.22	0.22	2.05	3.25	1.2	0.63	0.37	0.08	0.09	0.11	0.13	0.12	0.11	0.58	1.18	0.08	0.29	0.68	0.4	0.88	-0.1	4	2	-0.1	-6.1	0.11
137	222	MY	45	m	-2	7	-4.1	4.05	-0.5	1.77	1.46	0.74	0.59	0.08	66	0.08	66	0.11	292	1.21	1.52	0.24	0.23	0.79	1.96	1.17	0.4	0.6	0.15	0.13	0.19	0.28	0.22	0.13	0.14	0.99	0.21	0.17	0.38	0.47	0.93	-0.1	5	3	-0	15.5	0.04
137	209	FI	70	f	1.38	10	-8.4	13.3	-0.2	1.52	1.56	0.68	0.72	0	280	0.3	60	0	280	0.98	0.92	0.49	0	0.92	1.89	0.97	0.49	0.51	0.15	0.31	0.31	0.2	0.04	0.01	0.23	0.38	0.2	0.25	0.64	0.44	0.78	-0.2	6	4	0.95	4.74	0.95
137	222	FI	58	f	-1.6	16	-6	13.2	-0.4	1.64	1.44	0.82	0.62	0.03	87	0.03	87	0.13	299	1.14	1.49	0.05	0.25	0.94	1.94	1	0.48	0.52	0.1	0.06	0.16	0.28	0.18	0.14	0.17	0.57	0.16	0.18	0.44	0.34	0.73	-0.3	5	3	-0.4	3.09	0.4
137	216	SS	67	m	-3.6	13	-6.6	12.2	0.05	1.93	1.72	0.9	0.76	0.01	46	0.04	72	0.01	295	1.13	1.33	0.09	0.12	1.65	2.58	0.93	0.64	0.36	0.04	0.05	0.17	0.2	0.14	0.04	0.21	0.98	0.2	0.13	0.33	0.56	0.72	-0.3	5	3	0.13	15.8	0.13
138	245	MY	48	m	-6.3	15	-5.8	7.74	-0.2	1.9	1.88	0.87	0.77	0	47	0.02	60	0.02	298	1.02	1.15	0.14	0.1	1.76	2.75	0.99	0.64	0.36	0.04	0.05	0.19	0.19	0.13	0.03	0.46	1.58	0.26	0.26	0.59	0.81	1.1	0.1	6	4	0.27	18	0.27
138	215	GE	71	m	-0.6	11	0.17	2.86	1.09	2.1	2.03	0.8	0.73	0.06	41	0.07	61	0.07	294	1.03	1.12	0.24	0.19	1.92	3.36	1.44	0.57	0.43	0.08	0.09	0.15	0.18	0.16	0.08	0.39	0.96	0.19	0.2	0.53	0.48	0.76	-0.2	4	2	0.05	7.18	0.05
138	208	GE	70	f	0.75	14	-2.2	1.33	-0.1	1.7	1.69	0.8	0.78	0.06	279	0.12	72	0.06	279	1.01	1.05	0.21	0.13	1.34	2.27	0.94	0.59	0.41	0.12	0.12	0.12	0.12	0.13	0.09	0.3	0.88	0.11	0.23	0.68	0.73	0.96	-0	5	3	0.14	13	0.14
138	245	GE	44	m	-1	18	0.3	1.25	0.19	1.84	1.82	0.9	0.85	0	312	0.01	61	0.01	299	1.01	1.08	0.11	0.07	2	2.64	0.64	0.76	0.24	0.02	0.03	0.12	0.13	0.09	0.02	0.49	1.06	0.06	0.25	0.61	0.34	0.85	-0.2	6	4	0.29	9.31	0.29
138	209	MY	48	m	-3.8	14	-5.9	12	-0	1.83	1.56	0.84	0.79	0	0	0.08	61	0	291	1.17	1.24	0.21	0.09	1.5	2.22	0.73	0.67	0.33	0.01	0.09	0.15	0.19	0.1	0.02	0.23	1.13	0.13	0.15	0.39	0.57	0.86	-0.1	7	5	0.69	12.6	0.69
138	224	GE	75	m	0	8	-8.7	13.1	-0.1	1.86	1.86	0.9	0.77	0.01	280	0.05	61	0.01	280	1	1.17	0.16	0.03	1.74	2.72	0.98	0.64	0.36	0.1	0.07	0.14	0.13	0.09	0.04	0.44	0.89	0.12	0.25	0.5	0.63	0.69	-0.3	6	4	0.23	-18	0.23
138	215	SS	63	f	-4.5	19	-1.1	6.1	-0.1	1.88	1.76	0.85	0.88	0	0	0.06	60	0	293	1.07	1.02	0.22	0.07	1.93	2.59	0.66	0.75	0.25	0.01	0.08	0.12	0.12	0.09	0.01	0.37	0.7	0.05	0.2	0.44	0.33	0.58	-0.4	7	5	0.76	-1.6	0.76
138	218	FI	63	m	-5.9	13	-0.4	4.14	0.17	1.7	1.52	0.81	0.61	0	287	0.17	61	0	287	1.12	1.49	0.3	0.03	0.89	2.09	1.21	0.42	0.58	0.14	0.17	0.21	0.28	0.26	0.03	0.16	1.22	0.28	0.18	0.34	0.65	0.99	-0	6	4	0.66	14.1	0.66
138	229	GE	73	f	-2.6	15	-3.6	3.15	0.16	1.7	1.43	0.8	0.79	0.07	270	0.09	70	0.07	270	1.19	1.21	0.21	0.13	1.23	1.98	0.75	0.62	0.38	0.08	0.1	0.15	0.13	0.09	0.08	0.33	0.88	0.07	0.27	0.72	0.4	1.03	0.03	5	3	0.13	2.55	0.13
138	217	FI	52	m	-4.5	14	-3.4	3.74	-0.5	1.83	2.15	0.75	0.71	0	281	0.2	60	0	281	0.85	0.9	0.42	0.03	1.59	3.05	1.47	0.52	0.48	0.12	0.21	0.22	0.17	0.13	0.01	0.37	0.65	0.19	0.24	0.55	0.39	0.69	-0.3	6	4	0.89	-5.5	0.89
138	207	GE	76	m	-0.9	13	-0.2	2.74	-0.1	1.93	1.93	0.92	0.84	0	281	0.04	60	0	281	1	1.1	0.12	0.04	2.05	2.83	0.78	0.73	0.28	0.03	0.05	0.13	0.14	0.11	0	0.51	1.19	0.06	0.25	0.76	0.33	1.02	0.02	7	5	0.88	7.97	0.88

138	221	GE	68	f	-2.1	12	-11	15.5	-1	1.75	1.72	0.98	0.74	0	49	0	60	0.01	257	1.02	1.36	0.02	0.02	1.68	2.32	0.64	0.72	0.28	0.01	0	0.09	0.26	0.08	0.01	0.22	0.65	0.04	0.13	0.5	0.34	0.72	-0.3	8	6	-0.6	2.35	0.62
138	224	SS	74	m	-2	11	-6.4	12.4	-0.1	1.84	1.97	0.91	0.76	0	254	0.05	62	0	254	0.94	1.13	0.15	0.01	1.97	2.88	0.91	0.69	0.31	0.03	0.06	0.14	0.21	0.05	0.01	0.31	0.88	0.05	0.16	0.4	0.36	0.63	-0.4	7	5	0.83	0.17	0.83
139	223	MY	63	f	-10	17	-8.6	10.1	-0.2	1.89	1.61	0.9	0.77	0	6	0.04	62	0.02	299	1.18	1.38	0.08	0.11	1.47	2.34	0.87	0.63	0.37	0.01	0.04	0.18	0.23	0.2	0.04	0.26	0.4	0.02	0.18	1.05	0.54	1.1	0.1	7	5	-0	0	0.05
139	221	GE	63	m	0.25	11	-1.6	6.23	0.26	2.23	2.1	0.87	0.82	0	321	0.06	61	0.01	297	1.06	1.12	0.2	0.1	2.66	3.67	1.01	0.73	0.28	0.01	0.07	0.11	0.15	0.1	0.02	0.8	1.82	0.15	0.3	0.67	0.41	0.99	-0	7	5	0.59	9.16	0.59
139	192	MY	61	m	-5	11	-4.1	6.6	0.03	1.76	1.49	0.79	0.77	0	38	0.05	60	0.03	293	1.18	1.21	0.14	0.22	1.1	1.98	0.88	0.56	0.45	0.03	0.08	0.2	0.23	0.24	0.07	0.13	0.81	0.21	0.12	0.43	0.76	0.88	-0.1	6	4	0.14	27	0.14
139	252	SS	53	m	-4.8	16	-11	15.7	-0.6	1.93	2.19	0.89	0.66	0.02	0	0.06	60	0.03	284	0.88	1.18	0.13	0.09	1.78	3.3	1.52	0.54	0.46	0.04	0.06	0.15	0.31	0.24	0.04	0.25	0.63	0.39	0.15	0.41	0.91	0.65	-0.4	5	3	0.21	17.6	0.21
139	212	MY	59	m	-3.9	16	-6	12.3	0.04	1.83	1.86	0.97	0.75	0	21	0	60	0	275	0.98	1.28	0.06	0	1.89	2.62	0.74	0.72	0.28	0.01	0.01	0.1	0.23	0.12	0	0.22	0.7	0.1	0.11	0.42	0.34	0.65	-0.4	8	6	0.75	6.54	0.75
139	225	FI	64	f	-0.8	10	-1.6	3.41	-0.2	1.5	1.56	0.81	0.73	0.02	272	0.15	79	0.02	272	0.96	1.08	0.23	0.06	1.01	1.81	0.8	0.56	0.44	0.13	0.16	0.16	0.16	0.08	0.05	0.21	0.71	0.1	0.21	0.5	0.45	0.83	-0.2	5	3	0.47	2.26	0.47
139	220	MY	50	f	-5	13	-6	13.2	0.51	1.68	1.45	0.71	0.66	0.01	298	0.2	61	0.01	298	1.16	1.24	0.35	0.15	0.86	1.81	0.95	0.48	0.52	0.1	0.2	0.25	0.24	0.13	0.04	0.18	0.64	0.28	0.22	0.48	1.05	0.87	-0.1	6	4	0.69	30.8	0.69
139	200	MY	69	f	-4.5	11	-10	14.4	0.08	1.86	1.6	0.83	0.77	0.01	291	0.1	92	0.01	291	1.17	1.25	0.2	0.13	1.41	2.34	0.93	0.6	0.4	0.04	0.12	0.18	0.21	0.13	0.03	0.15	1.4	0.29	0.11	0.34	0.96	1	-0	6	4	0.65	23.1	0.65
140	207	SS	71	f	2.75	13	-2.1	2.48	-0.7	1.87	1.86	0.84	0.76	0	257	0.14	120	0	257	1.01	1.11	0.3	0	1.71	2.73	1.03	0.63	0.38	0.11	0.17	0.15	0.13	0.04	0.03	0.33	1.2	0.09	0.2	0.54	0.47	0.9	-0.1	6	4	0.71	8.28	0.71
140	275	FI	63	m	0.88	8	-1.1	9.34	-0.4	1.82	1.86	0.72	0.6	0.02	58	0.02	62	0.19	295	0.98	1.18	0.13	0.39	1.1	2.72	1.63	0.4	0.6	0.13	0.06	0.21	0.27	0.26	0.2	0.2	0.77	0.23	0.18	0.53	0.38	0.82	-0.2	5	3	-0.6	2.83	0.55
140	232	MY	31	m	-3.9	18	-2.4	6.17	0.29	1.86	1.42	0.83	0.87	0	38	0.02	62	0.07	284	1.31	1.25	0.14	0.17	1.44	2.05	0.61	0.71	0.3	0.04	0.04	0.09	0.1	0.14	0.09	0.25	0.35	0.05	0.19	0.51	0.3	0.58	-0.4	6	4	-0.4	2.6	0.4
140	207	FI	67	f	-0.4	16	-7	13.2	-0.9	1.88	1.82	0.89	0.83	0	254	0.09	60	0	254	1.03	1.11	0.19	0.01	2	2.71	0.71	0.74	0.26	0.05	0.09	0.11	0.13	0.02	0.02	0.42	0.45	0.04	0.27	0.58	0.45	0.59	-0.4	6	4	0.68	-1	0.68
140	223	MY	58	f	-1.9	15	-11	17.5	0.2	1.64	1.51	0.68	0.68	0.01	303	0.22	82	0.02	299	1.09	1.08	0.37	0.17	0.8	1.8	1	0.44	0.56	0.1	0.22	0.25	0.24	0.2	0.04	0.11	0.46	0.29	0.14	0.2	0.71	0.53	-0.5	5	3	0.75	11.8	0.75
140	208	GE	67	f	-2.9	12	-4.7	10.4	-1.1	2.23	2.26	0.85	0.84	0.02	293	0.09	60	0.02	293	0.99	1	0.26	0.07	2.85	3.97	1.13	0.72	0.28	0.06	0.1	0.14	0.1	0.04	0.03	0.39	0.58	0.07	0.21	0.98	0.53	1.05	0.05	5	3	0.55	4.88	0.55
140	230	FI	56	f	-3.8	15	-0.9	6.02	-0.1	1.66	1.57	0.62	0.69	0.04	321	0.2	115	0.11	300	1.06	0.96	0.35	0.27	0.89	2.01	1.12	0.44	0.56	0.11	0.21	0.21	0.21	0.17	0.13	0.19	0.98	0.23	0.21	0.6	0.59	1.11	0.11	5	3	0.2	18.6	0.2
140	217	MY	56	f	-10	11	-9.3	13.6	-1.1	1.8	1.76	0.93	0.7	0	69	0	69	0	295	1.02	1.36	0.07	0.05	1.55	2.51	0.95	0.62	0.38	0.04	0.03	0.16	0.24	0.17	0.02	0.19	0.75	0.11	0.12	0.38	0.38	0.66	-0.3	6	4	0.13	4.21	0.13
140	245	GE	67	f	0.13	16	-6	10.3	-0.5	1.89	1.5	0.9	0.67	0.02	81	0.02	81	0.07	275	1.27	1.69	0.05	0.14	1.14	2.25	1.11	0.51	0.49	0.18	0.07	0.17	0.16	0.15	0.13	0.19	1.09	0.24	0.17	0.34	0.27	0.86	-0.1	5	3	-0.3	8.63	0.27
140	232	FI	64	f	-2	17	-6.3	10.7	-0.6	1.63	1.72	0.73	0.69	0	53	0	60	0.16	299	0.95	1.01	0.15	0.29	0.96	2.13	1.18	0.45	0.55	0.07	0.05	0.21	0.26	0.31	0.19	0.18	0.52	0.18	0.19	0.59	0.52	0.82	-0.2	6	4	-0.7	4.61	0.66
141	192	GE	63	f	1.25	15	-1	2.04	-0.1	2.06	2.02	0.83	0.82	0.01	263	0.15	62	0.01	263	1.02	1.03	0.33	0.02	2.22	3.28	1.06	0.68	0.32	0.08	0.15	0.16	0.11	0.03	0.02	0.54	1.16	0.07	0.25	0.74	0.73	0.95	-0.1	6	4	0.76	6.07	0.76
141	248	FI	63	m	-0.3	15	0.03	3.06	0.01	1.78	1.89	0.83	0.76	0	260	0.17	120	0	260	0.94	1.03	0.34	-0	1.66	2.72	1.08	0.61	0.4	0.12	0.19	0.18	0.14	0.05	0	0.47	1.34	0.15	0.28	0.62	0.5	1	-0	7	5	0.96	14.5	0.96
141	186	GE	69	f	0.38	14	-3.1	5.09	-0.2	2.08	2.05	0.9	0.87	0	282	0.07	65	0	282	1.01	1.06	0.18	0.02	2.49	3.29	0.81	0.76	0.25	0.05	0.07	0.09	0.1	0.08	0.01	0.62	1.16	0.06	0.25	1.19	0.33	1.38	0.38	6	4	0.76	4.98	0.76
141	214	FI	58	f	-2	15	0.51	5.33	0.06	1.96	1.77	0.65	0.61	0.08	8	0.1	72	0.19	300	1.11	1.18	0.23	0.45	0.96	2.65	1.69	0.36	0.64	0.1	0.11	0.26	0.31	0.31	0.22	0.14	0.18	0.16	0.16	0.55	0.31	0.56	-0.4	5	3	-0.3	1.86	0.33
141	251	MY	55	f	-8.4	12	-8.7	14.3	-0.2	2.01	1.89	0.89	0.83	0	0	0.1	68	0	278	1.06	1.15	0.21	0.01	2.16	3.06	0.9	0.71	0.3	0.02	0.1	0.13	0.18	0.06	0	0.4	1.66	0.14	0.19	0.51	0.41	0.97	-0	8	6	0.96	7.57	0.96
141	271	MY	63	f	-6.4	17	-2.8	8.57	-0.6	1.94	1.57	0.67	0.65	0.11	308	0.17	120	0.11	298	1.23	1.27	0.37	0.27	0.95	2.29	1.34	0.42	0.59	0.15	0.2	0.18	0.21	0.21	0.13	0.2	0.9	0.23	0.21	0.5	0.59	0.89	-0.1	4	2	0.15	15.8	0.15
142	227	FI	71	f	0	12	-9.7	16.1	-0.3	1.74	1.55	0.75	0.62	0	55	0	60	0.18	297	1.12	1.36	0.07	0.37	1.03	2.12	1.1	0.48	0.52	0.17	0.02	0.11	0.21	0.23	0.19	0.21	0.59	0.16	0.2	0.48	0.55	0.73	-0.3	6	4	-0.8	10.6	0.81
142	192	GE	64	f	0.25	12	-5.9	6.21	-1.1	1.72	1.7	0.88	0.81	0.03	300	0.04	75	0.03	300	1.01	1.1	0.09	0.12	1.65	2.31	0.66	0.72	0.28	0.06	0.05	0.08	0.12	0.09	0.04	0.47	1.28	0.08	0.28	0.49	0.51	0.88	-0.1	5	3	0.1	9.24	0.1
142	196	FI	56	f	0.13	14	-2.4	9.19	-0.3	1.7	1.57	0.81	0.74	0.02	66	0.02	66	0.12	264	1.09	1.19	0.11	0.22	1.17	2.1	0.93	0.56	0.44	0.14	0.05	0.13	0.13	0.15	0.15	0.24	0.85	0.08	0.2	0.49	0.29	0.82	-0.2	5	3	-0.5	3.56	0.54
142	188	GE	55	m	1.75	12	-5.7	11.9	0.07	1.73	1.6	0.92	0.78	0	55	0.01	60	0.01	280	1.08	1.27	0.1	0.04	1.34	2.09	0.75	0.64	0.36	0.04	0.06	0.14	0.19	0.2	0.03	0.42	1.18	0.14	0.31	0.85	0.35	1.28	0.28	6	4	0.39	8.89	0.39
142	202	MY	51	f	-7.6	14	-1.9	6.14	-0.2	1.81	1.41	0.7	0.66	0.06	316	0.14	60	0.08	299	1.29	1.36	0.33	0.22	0.92	1.96	1.04	0.47	0.53	0.08	0.16	0.23	0.25	0.17	0.1	0.13	0.66	0.18	0.14	0.35	0.53	0.71	-0.3	5	3	0.24	15	0.24
142	199	SS	53	m	-0.6	16	-1.5	3.81	0.08	1.75	1.92	0.76	0.84	0.05	12	0.11	119	0.08	300	0.92	0.83	0.23	0.18	1.7	2.59	0.89	0.66	0.34	0.07	0.12	0.11	0.1	0.11	0.09	0.34	0.73	0.05	0.2	0.46	0.37	0.62	-0.4	5	3	0.12	-6.7	0.12
142	242	MY	53	m	-4.8	10	0.51	1.62	-0.3	2.06	1.76	0.69	0.69	0.07	309	0.17	60	0.07	298	1.17	1.17	0.38	0.27	1.29	2.78	1.49	0.47	0.54	0.12	0.12	0.17	0.18	0.2	0.09	0.18	0.53	0.13	0.14	0.4	0.38	0.57	-0.4	4	2	0.32	5.68	0.32
143	209	MY	68	f	-1	15	0.07	1.39	-0.6	1.8	1.39	0.82	0.77	0	15	0.05	60	0.01	293	1.29	1.38	0.24	0.09	1.06	1.92	0.86	0.55	0.45	0.01	0.08	0.23	0.24	0.27	0.06	0.21	0.87	0.26	0.2	0.47	0.48	0.96	-0	7	5	0.13	16	0.13
143	220	FI	54	f	-1.1	12	-0.8	3.92	0.06	1.65	1.33	0.81	0.63	0.02	280	0.12	60	0.02	280	1.24	1.59	0.27	0.04	0.82	1.71	0.89	0.48	0.52	0.09	0.14	0.19	0.3	0.23	0.04	0.09	0.65	0.15	0.11	0.31	0.62	0.73	-0.3	5	3	0.56	16.7	0.56
143	242	FI	71	f	-1.4	15	0.02	4.05	0.13	1.9	1.66	0.78	0.76	0.02	335	0.12	60	0.04	297	1.15	1.17	0.31	0.12	1.43	2.5	1.07	0.57	0.43	0.04	0.13	0.2	0.22	0.13	0.05	0.24	0.47	0.07	0.2	0.57	0.35	0.68	-0.3	5	3	0.46	-7.5	0.46
143	182	GE	63	f	1.25	14	-1.1	2.22	-0.1	1.97	2.02	0.71	0.81	0.07	354	0.17	120	0.09	247	0.98	0.86	0.37	0.19	1.87	3.19	1.32	0.59	0.41	0.1	0.18	0.17	0.11	0.09	0.1	0.3	0.74	0.1	0.16	0.37	0.3	0.54	-0.5	5	3	0.31	-4	0.31
144	217	FI	69	f	-1	14	-1.6	2.02	-0.5	1.38	1.5	0.87	0.69	0	260	0.11	65	0	260	0.92	1.17	0.18	-0	0.92	1.65	0.73	0.56	0.45	0.08	0.12	0.27	0.26	0.04	0.01	0.12	0.46	0.11	0.13	0.18	0.5	0.46	-0.5	7	5	0.85	5.7	0.85
144	180	FI	74	m	-0.4	15	-2.9	9.41	-0.2	1.76	1.57	0.84	0.73	0.01	287	0.13	60	0.01	287	1.12	1.27	0.27	0.02	1.35	2.18	0.83	0.62	0.38	0.09	0.13	0.16	0.16	0.07	0.02	0.2	1.02	0.14	0.15	0.47	0.46	0.92	-0.1	6	4	0.72	7.06	0.72
144	230	MY	71	f	1.38	12	-8.3	12	-0.2	1.62	1.57	0.86	0.53	0.04	64	0.04	64	0.05	297	1.03	1.68	0.09	0.14	0.83	1.96	1.13	0.42	0.58	0.09	0.08	0.22	0.38	0.29	0.09	0.17	0.85	0.29	0.21	0.61	0.67	1.1	0.1	5	3	-0.1	21	0.09
145	214	MY	62	m	-3.5	12	-3.2	2.62	0.27	1.83	1.58	0.76	0.77	0.04	321	0.06	73	0.07	299	1.16	1.14	0.15	0.3	1.29	2.21	0.91	0.59	0.41	0.08	0.09	0.14	0.14	0.17	0.1	0.39	1.06	0.16	0.3	0.65	0.49	1.03	0.03	5	3	-0	16.6	0.02
145	176	GE	64	m	-0.6	11	-2.6	3.37	-0	2.01	1.72	0.81	0.76	0.01	294	0.13	60	0.01	294	1.17	1.25	0.3	0.08	1.59	2.64	1.05	0.6	0.4	0.06	0.14	0.17	0.17	0.13	0.02	0.39	1.72	0.21	0.24	0.67	0.72	1.26	0.26	5	3	0.72	12.6	0.72
145	271	MY	49	f	-3.4	17	-1.5	1.83	0.47	2.07	1.47	0.71	0.77	0	0	0.17	83	0.02	295	1.41	1.31	0.37	0.22	1.15	2.34	1.2	0.49	0.51	0.02	0.17	0.2	0.25	0.27	0.08	0.26	0.8	0.4	0.23	0.42	0.79	0.81	-0.2	7	5	0.45	25.8	0.45
145	225	MY	70	f	2.13	14	-0.6	2.19	-0.1	1.72	1.53	0.73	0.72	0.04	288	0.17	60	0.04	288	1.13	1.14	0.34	0.12	1.06	2.08	1.02	0.51	0.49	0.1	0.18	0.21	0.19	0.15	0.06	0.29	0.76	0.21	0.28	1.07	0.63	1.37	0.37	5	3	0.51	16.5	0.51
145	241	MY	42	m	0	10	-11	11.5	-0.4	1.75	1.37	0.89	0.82	0	0	0.01	62	0.02	283	1.27	1.39	0.14	0.05	1.31	1.9	0.59	0.69	0.31	0.01	0.05	0.16	0.19	0.11	0.03	0.28	1.12	0.11	0.21	0.58	0.44	1.08	0.08	7	5	0.31	14.7	0.31
146	209	MY	67	f	-8.5	10	-6.8	9.05	-0.4	1.76	1.46	0.86	0.81	0	0	0.04	60	0	299	1.21	1.28	0.19	0.05	1.42	2.01	0.6	0.71	0.3	0	0.08	0.18	0.18	0.08	0.01	0.1	1.2	0.06	0.07	0.48	0.43	1.09	0.09	8	6	0.81	10.8	0.81
146	249	MY	59	f	-9.6	16	-6.7	7.6	-0.8	1.85	1.13	0.74	0.71	0	0	0.07	60	0	291	1.64	1.7	0.29	0.2	0.83	1.59	0.77	0.52	0.48	0	0.12	0.22	0.28	0.23	0.06	0.13	1.13	0.3	0.16	0.4	1.11	1.24	0.24	8	6	0.4	42.4	0.4
147	217	FI	56	f	-1.1	16	-6.3	9.34	-1.4	1.87	1.68	0.78	0.68	0	295	0.15	60	0	295	1.11	1.26	0.34	0.08	1.37	2.36	0.99	0.58	0.42	0.08	0.16	0.19	0.2	0.09	0.03	0.33	0.5	0.12	0.26	0.68	0.26	0.79	-0.2	6	4	0.71	-1.3	0.71
147	211	FI	68	f	-1.8	14	-3.4	5.69	-0.1	1.53	1.49	0.83	0.6	0.04	254	0.11	96	0.04	254	1.03	1.41	0.17	0.09	0.81	1.72	0.91	0.47	0.53	0.21	0.17	0.13	0.17	0.07	0.15	0.09	0.27	0.07	0.11	0.41	0.33	0.56	-0.4	5	3	0.06	-1.4	0.06
147	234	MY	60	m	-2.6	14	-11	14	0.18	1.92	1.7	0.92	0.71	0	112	0	112	0.02	293	1.13	1.47	0.02	0.13	1.63	2.55	0.91	0.64	0.36	0.09	0.03	0.09	0.19	0.14	0.05	0.42	1.77	0.15	0.26	0.59	0.46	1.19	0.19	6	4	-0.3	6.2	0.3
147	215	MY	40	m	-7.5	14	-2.7	2.36	-0	2.17	1.88	0.83	0.63	0.02	314	0.07	62	0.06	299	1.15	1.52	0.24	0.14	1.37	2.66	1.28	0.52	0.48	0.03	0.12	0.21	0.26	0.2	0.09	0.25	0.89	0.34	0.19	0.56	0.77	0.94	-0.1	5	3	0.07	21	0.07
147	237	MY	67	m	-3.3	13	-1.2	2.84	-0	2.17	1.85	0.92	0.78	0	67	0	67	0.01	288	1.18	1.4	0.06	0.11	2.08	3.1	1.01	0.67	0.33	0.02	0.03	0.15	0.19	0.15	0.03	0.49	1.65	0.22	0.24	0.54	0.66	1	0	6	4	0.06	17.4	0.06
147	207	FI	60	f	-4.3	12	-8.8	11.9	0.02	1.96	1.86	0.78	0.84	0.01	335	0.12	102	0.05	300	1.05	0.98	0.23	0.2	1.71	2.85	1.14	0.6	0.4	0.08	0.13	0.12	0.14	0.17	0.07	0.39	0.91	0.24	0.23	0.43	1.09	0.67	-0.3	5	3	0.34	16.3	0.34
147	193	SS	74	m	0.75	12	-4.1	6.65	0.05	1.51	1.5	0.83	0.73	0.01	17	0.03	61	0.08	299	1.01	1.14	0.07	0.2	1.07	1.8	0.74	0.59	0.41	0.03	0.03	0.13	0.24	0.21	0.1	0.13	0.46	0.06	0.12	0.22	0.44	0.44	-0.6	6	4	-0.5	3.97	0.5
147	246	FI	53	f	-0.1	15	-1.1	1.13	0.03	1.66	1.5	0.75	0.66	0.01	297	0.18	62	0.01	297	1.1	1.26	0.34	0.07	0.91	1.97	1.06	0.46	0.54	0.13	0.19	0.22	0.22	0.21	0.04	0.15	0.91	0.15	0.16	0.45	0.39	0.92	-0.1	5	3	0.62	-0.7	0.62
148	199	MY	52	m	-9.8	14	-4.3	9.98	0.03	1.75	1.13	0.86	0.71	0	23	0	73	0.04	300	1.56	1.89	0.04	0.21	0.82	1.51	0.68	0.55	0.45	0.01	0.02	0.18	0.29	0.29	0.12	0.12	0.94	0.19	0.15	0.4	0.78	1.08	0.08	7	5	-0.7	30.7	0.72
148	257	MY	37	m	-5	10	-3.9	11.1	-0.6	1.93	1.74	0.86	0.79	0.01	283	0.11	75	0.01	283	1.11	1.21	0.24	0.04	1.53	2.59	1.06	0.59	0.41	0.06	0.12	0.22	0.18	0.14	0.02	0.26	0.97	0.2	0.17	0.49	0.52	0.84	-0.2	6	4	0.69	13.6	0.69
148	214	MY	51	f	-5.5	12	-11	14.1	-0.6	1.43	1.15	0.86	0.72	0.03	284	0.05	70	0.03	284	1.24	1.5	0.13	0.07	0.71	1.27	0.57	0.56	0.45	0.08	0.06	0.16	0.22	0.2	0.06	0.09	0.56	0.12	0.12	0.28	0.65	0.74	-0.3	6	4	-0	26	0.03
148	205	MY	43	f	-7.3	13	-4.7	5.54	-0.4	1.78	1.08	0.73	0.68	0.02	46	0.06	61	0.06	300	1.66	1.77	0.28	0.2	0.65	1.47	0.82	0.44	0.56	0.05	0.12	0.28	0.27	0.29	0.08	0.06	0.47	0.17	0.09	0.33	0.68	0.74	-0.3	5	3	0.22	27.3	0.22
149	220	MY	50	m	-4.4	14	-5	8.97	-0.1	1.92	1.7	0.79	0.73	0.03	0	0.06	60	0.09	273	1.13	1.23	0.21	0.19	1.35	2.53	1.18	0.53	0.47	0.05	0.08	0.18	0.24	0.19	0.11	0.4	1.43	0.22	0.3	0.64	0.41	1.14	0.14	5	3	-0.1	8.7	0.12
149	213	MY	51	f	-4.8	12	-4.9	8.91	-0.7	2.29	1.85	0.92	0.8	0.01	68	0.01	68	0.02	275	1.24	1.42	0.14	0.05	2.2	3.27	1.07	0.67	0.33	0.03	0.03	0.15	0.2	0.13	0.03	0.39	1.64	0.28	0.18	0.51	0.74	0.99	-0	6	4	0	20	0
149	244	MY	55	f	-8.8	14	-3.2	8.45	0.24	1.95	1.93	0.77	0.75	0.05	63	0.05	63	0.07	300	1.01	1.03	0.22	0.23	1.6	2.89	1.3	0.55	0.45	0.07	0.09	0.21	0.19	0.15	0.08	0.47	1.45	0.24	0.29	0.87	0.55	1.29	0.29	5	3	0.05	12.5	0.05
149	204	FI	58	f	-1	13	-2.6	3.96	-0.1	1.89	1.75	0.7	0.69	0.09	70	0.09	70	0.17	298	1.08	1.09	0.23	0.35	1.03	2.55	1.53	0.4	0.6	0.16	0.16	0.27	0.19	0.18	0.17	0.13	1.21	0.26	0.13	0.3	0.32	0.83	-0.2	5	3	-0.1	0.11	0.06
149	199	MY	69	f	-3	13	-2.7	5.92	-0.3	1.42	1.32	0.76	0.71	0.02	276	0.18	60	0.02	276	1.07	1.16	0.3	0.04	0.76	1.46	0.7	0.52	0.48	0.05	0.19	0.28	0.27	0.12	0.02	0.14	0.34	0.15	0.19	0.31	0.55	0.54	-0.5	6	4	0.79	17.4	0.79
150	256	MY	49	m	-8	18	-8.2	14.3	-0.4	1.78	1.81	0.86	0.71	0.04	292	0.07	63	0.04	292	0.99	1.2	0.15	0.09	1.47	2.47	1	0.6	0.4	0.08	0.09	0.15	0.19	0.1	0.05	0.27	1.38	0.16	0.19	0.44	0.61	0.96	-0	5	3	0.35	6.42	0.35
150	200	FI	59	f	1	15	-3.9	7.68	-0.5	1.72	1.59	0.81	0.73	0.01	278	0.17	61	0.01	278	1.09	1.2	0.31	0.02	1.23	2.13	0.9	0.58	0.42	0.12	0.18	0.18	0.17	0.07	0.02	0.22	0.65	0.1	0.18	0.52	0.38	0.77	-0.2	6	4	0.76	7.29	0.76
151	178	FI	72	m	-0.3	14	-4.3	12	0.07	1.63	1.46	0.85	0.8	0.01	299	0.12	80	0.01	299	1.12	1.19	0.22	0.03	1.16	1.87	0.71	0.62	0.38	0.06	0.13	0.15	0.16	0.16	0.02	0.21	0.39	0.06	0.18	0.42	0.16	0.55	-0.5	5	3	0.75	3.45	0.75
151	204	SS	64	m	-1.6	16	-0.5	2.54	0.22	2.09	1.75	0.79	0.71	0.07	286	0.13	94	0.07	286	1.19	1.33	0.28	0.16	1.57	2.97	1.41	0.53	0.47	0.12	0.15	0.14	0.19	0.14	0.08	0.32	0.93	0.16	0.23	0.6	0.58	0.88	-0.1	4	2	0.33	12.1	0.33
151	223	SS	75	f	1.63	12	-0.3	2.93	0.05	2.03	2.01	0.79	0.7	0	285	0.17	60	0	285	1.01	1.14	0.42	0.01	1.73	3.14	1.41	0.55	0.45	0.08	0.18	0.21	0.23	0.12	0.01	0.27	0.38	0.06	0.17	0.47	0.35	0.5	-0.5	6	4	0.94	-6.8	0.94
151	209	MY	60	f	-5.5	13	-7.8	10.9	-0.2	1.98	1.4	0.9	0.82	0	282	0.01	64	0	282	1.41	1.54	0.09	0.12	1.23	1.98	0.76	0.62	0.38	0.02	0.05	0.23	0.21	0.2	0.02	0.1	0.97	0.14	0.08	0.19	0.6	0.7	-0.3	7	5	0.51	12.6	0.51

151	238	MY	75	m	0.88	10	-1.3	3.53	0.07	1.95	1.81	0.83	0.76	0	281	0.12	60	0	281	1.08	1.17	0.32	0.02	1.76	2.72	0.96	0.65	0.35	0.02	0.13	0.17	0.21	0.08	0.01	0.34	1.09	0.17	0.19	0.44	0.57	0.79	-0.2	7	5	0.89	13.2	0.89
151	217	GE	45	f	0	11	-6.5	11.2	-0.4	1.71	1.58	0.83	0.84	0	309	0.05	61	0.02	300	1.08	1.08	0.17	0.11	1.48	2.13	0.66	0.69	0.31	0.06	0.07	0.13	0.11	0.12	0.04	0.5	1.27	0.08	0.34	0.72	0.26	1.12	0.12	6	4	0.2	2.1	0.2
151	227	MY	63	m	-4.5	13	-1.8	8.28	0.07	1.84	1.42	0.71	0.81	0	7	0.22	87	0.01	291	1.3	1.15	0.4	0.13	1.02	1.98	0.97	0.51	0.49	0.03	0.22	0.24	0.21	0.21	0.05	0.22	0.9	0.3	0.22	0.36	0.8	0.86	-0.1	6	4	0.63	28.4	0.63
152	250	GE	62	f	0.38	17	-1.9	1.74	-0.2	1.65	1.76	0.82	0.8	0.04	314	0.07	68	0.06	300	0.94	0.96	0.15	0.16	1.54	2.29	0.75	0.67	0.33	0.09	0.08	0.1	0.1	0.1	0.07	0.24	0.97	0.03	0.16	0.54	0.44	0.87	-0.1	5	3	0.03	-2.8	0.03
152	217	MY	66	f	-6.3	17	-3.3	10.1	0.18	1.97	2.04	0.81	0.78	0.04	298	0.09	60	0.04	298	0.96	1	0.25	0.13	1.83	3.12	1.29	0.59	0.41	0.08	0.09	0.15	0.18	0.13	0.05	0.41	1.38	0.29	0.22	1	0.96	1.41	0.41	5	3	0.26	20.6	0.26
152	229	FI	70	m	-0.3	12	0.28	1.56	1.11	1.9	1.82	0.72	0.76	0.07	12	0.08	69	0.14	298	1.05	0.99	0.21	0.31	1.51	2.71	1.2	0.56	0.44	0.1	0.1	0.12	0.14	0.18	0.14	0.37	1.13	0.18	0.24	0.68	0.45	1.02	0.02	5	3	-0.2	8.96	0.22
153	234	FI	49	f	-2.5	14	-4.9	13.7	-0.3	1.71	1.69	0.83	0.67	0	277	0.14	62	0	277	1.01	1.26	0.26	0.03	1.25	2.25	1	0.56	0.44	0.06	0.14	0.18	0.26	0.14	0.01	0.33	0.85	0.18	0.26	0.79	0.54	1.11	0.11	6	4	0.92	9.97	0.92
153	254	MY	66	f	-14	18	-2.3	7.48	0.28	2.28	1.37	0.84	0.8	0	0	0	62	0.01	299	1.66	1.75	0.25	0.12	1.47	2.5	1.04	0.59	0.42	0	0.07	0.26	0.21	0.18	0.05	0.22	1.08	0.24	0.15	0.39	0.69	0.84	-0.2	8	6	0.04	16.8	0.04
154	222	GE	68	f	-5.8	10	-2.5	3.4	-0.1	1.92	1.75	0.98	0.76	0	78	0	78	0.01	275	1.09	1.41	0.03	0.01	1.85	2.63	0.77	0.71	0.29	0.08	0.02	0.1	0.14	0.07	0.04	0.46	0.81	0.05	0.25	0.48	0.33	0.63	-0.4	6	4	-0.4	-4.9	0.38
154	218	GE	59	m	1.25	14	-7.6	8.08	-0.3	2.19	2.17	0.86	0.88	0.02	133	0.02	120	0.1	298	1.01	0.99	0.09	0.22	2.75	3.73	0.97	0.74	0.26	0.07	0.06	0.03	0.07	0.11	0.1	1.03	1.84	0.08	0.37	0.71	0.44	0.95	-0.1	5	3	-0.2	1.95	0.24
154	241	MY	56	m	-7.5	14	-1.9	2.34	0.18	2.4	1.86	0.81	0.74	0	287	0.11	60	0	287	1.29	1.41	0.29	0.16	1.86	3.33	1.47	0.56	0.44	0.02	0.11	0.19	0.25	0.23	0.02	0.53	2.16	0.69	0.28	0.79	1.38	1.51	0.51	6	4	0.72	34.1	0.72
155	231	FI	40	m	-0.6	15	-3.5	8.25	-0.2	1.78	1.58	0.85	0.69	0	271	0.14	77	0	271	1.12	1.39	0.27	0	1.18	2.25	1.07	0.52	0.48	0.14	0.15	0.17	0.19	0.1	0.06	0.22	1.08	0.18	0.19	0.46	0.34	0.93	-0.1	6	4	0.41	9.11	0.41
155	233	GE	70	m	0.5	13	-1.2	1.98	0.2	1.74	1.57	0.94	0.74	0.03	80	0.03	80	0.03	272	1.11	1.4	0.05	0.06	1.35	2.12	0.77	0.64	0.36	0.1	0.07	0.11	0.15	0.08	0.07	0.39	0.79	0.08	0.29	0.73	0.34	0.97	-0	5	3	0.05	3.41	0.05
155	250	MY	46	f	-7.1	14	-3.8	4.66	0.13	1.87	1.65	0.9	0.81	0	74	0	74	0.02	300	1.13	1.26	0.03	0.16	1.51	2.37	0.86	0.64	0.36	0.03	0.02	0.16	0.18	0.2	0.08	0.27	0.55	0.07	0.19	0.56	0.26	0.72	-0.3	6	4	-0.6	-2.7	0.64
156	237	GE	43	m	0	15	1.87	1.08	0.14	2.08	1.99	0.78	0.8	0.05	328	0.1	62	0.07	300	1.04	1.02	0.31	0.15	2.07	3.16	1.1	0.65	0.35	0.06	0.11	0.13	0.13	0.08	0.07	0.58	1.8	0.19	0.28	0.54	0.43	0.99	-0	5	3	0.19	8.01	0.19
156	229	MY	58	f	-8.8	9	-1.9	3.08	-0.1	1.67	1.54	0.77	0.72	0.02	24	0.04	62	0.07	300	1.09	1.16	0.19	0.19	0.94	1.93	0.99	0.49	0.51	0.03	0.06	0.2	0.26	0.34	0.15	0.09	0.56	0.21	0.09	0.37	0.75	0.75	-0.3	5	3	-0.5	20.3	0.47
156	234	FI	76	f	0.75	16	-6.4	10.5	0.07	2.06	1.83	0.84	0.69	0.01	54	0.01	62	0.08	300	1.12	1.37	0.1	0.23	1.7	2.96	1.26	0.58	0.43	0.02	0.03	0.14	0.3	0.22	0.09	0.54	1.06	0.09	0.32	0.68	0.26	0.89	-0.1	5	3	-0.5	-1.9	0.52
157	222	FI	73	f	1.25	14	-4.3	6.25	-0.1	1.55	1.41	0.86	0.75	0	290	0.12	107	0	290	1.1	1.27	0.2	0.01	1.01	1.72	0.71	0.59	0.41	0.12	0.17	0.13	0.15	0.13	0.01	0.2	0.9	0.09	0.2	0.52	0.47	0.97	-0	6	4	0.86	15.8	0.86
157	245	MY	57	f	-8.9	12	-1.7	5.83	0.07	2.07	1.93	0.82	0.72	0	280	0.13	60	0	280	1.07	1.21	0.31	0.07	1.85	3.12	1.27	0.59	0.41	0.13	0.14	0.16	0.14	0.09	0.01	0.43	1.1	0.23	0.24	0.75	0.7	1.02	0.02	6	4	0.85	9.78	0.85
157	209	MY	64	m	-4.8	10	-4.4	8.54	-0	1.96	1.5	0.85	0.62	0	333	0.04	69	0	289	1.31	1.8	0.14	0.16	1.15	2.27	1.13	0.5	0.5	0.04	0.08	0.22	0.34	0.28	0.04	0.14	0.9	0.38	0.12	0.28	0.68	0.78	-0.2	6	4	0.35	21.1	0.35
157	215	MY	67	f	-5	14	-7.1	13.4	-0.1	2.13	1.93	0.89	0.66	0	285	0.1	81	0	285	1.1	1.48	0.21	0.02	1.89	3.3	1.41	0.57	0.43	0.06	0.11	0.17	0.28	0.1	0.02	0.21	0.54	0.15	0.12	0.39	0.56	0.53	-0.5	6	4	0.72	7.48	0.72
158	238	GE	65	m	-3.8	17	-1.8	2.47	0.23	1.94	2.22	0.93	0.75	0.02	256	0.05	95	0.02	256	0.87	1.07	0.09	0.05	2.25	3.42	1.17	0.66	0.34	0.12	0.08	0.07	0.11	0.03	0.09	0.74	1	0.12	0.34	0.82	0.41	0.92	-0.1	5	3	-0.1	-3.4	0.07
159	233	GE	75	m	1.38	17	-1	1.85	-0.1	1.71	1.66	0.79	0.75	0.05	322	0.12	61	0.06	298	1.03	1.1	0.21	0.14	1.3	2.23	0.93	0.58	0.42	0.06	0.12	0.15	0.19	0.13	0.07	0.25	0.5	0.13	0.24	0.4	0.77	0.57	-0.4	5	3	0.23	2.02	0.23
159	255	FI	56	f	-7.9	17	-5.8	13.5	-0	1.97	1.74	0.86	0.74	0.01	321	0.06	61	0.02	279	1.13	1.32	0.24	0.04	1.67	2.66	0.99	0.63	0.37	0.04	0.08	0.2	0.2	0.13	0.02	0.33	1.14	0.13	0.2	0.49	0.41	0.86	-0.1	5	3	0.54	0.92	0.54
159	239	MY	57	f	-3.8	16	-2.8	2.8	-0.5	1.73	1.5	0.57	0.53	0.05	14	0.17	60	0.2	299	1.16	1.24	0.33	0.42	0.62	2.09	1.47	0.3	0.7	0.08	0.19	0.3	0.39	0.34	0.24	0.1	0.8	0.44	0.16	0.31	0.87	0.85	-0.2	5	3	-0.1	24.5	0.1
159	204	FI	65	m	-0.9	12	-8.5	14.2	-0.9	1.99	1.72	0.85	0.74	0.03	61	0.03	61	0.06	284	1.16	1.32	0.17	0.14	1.68	2.71	1.04	0.62	0.38	0.13	0.05	0.13	0.11	0.11	0.09	0.26	0.95	0.11	0.15	0.39	0.43	0.68	-0.3	5	3	-0.3	10.9	0.28
160	246	MY	51	f	-7.8	10	-10	14.3	0.19	2	1.65	0.97	0.8	0	65	0	65	0.02	276	1.21	1.47	0.02	0.04	1.74	2.53	0.79	0.69	0.31	0.05	0.01	0.14	0.16	0.12	0.04	0.22	1.15	0.07	0.13	0.57	0.41	0.98	-0	6	4	-0.6	10.4	0.6
160	254	MY	61	m	-8.1	16	-6.1	12.9	-0.4	1.75	1.52	0.91	0.69	0.01	297	0.05	85	0.01	297	1.15	1.51	0.09	0.07	1.09	2.07	0.98	0.53	0.47	0.1	0.09	0.14	0.24	0.25	0.04	0.14	0.89	0.13	0.13	0.65	0.51	1.08	0.08	6	4	0.34	13.5	0.34
161	240	MY	53	m	-2.5	12	-2.6	3.2	-0.3	1.77	1.64	0.85	0.7	0.03	356	0.06	61	0.04	284	1.07	1.31	0.11	0.15	1.2	2.23	1.04	0.54	0.46	0.05	0.09	0.21	0.26	0.21	0.06	0.21	1.33	0.28	0.17	0.57	0.91	1.21	0.21	5	3	0.25	24.2	0.25
161	249	GE	64	m	-0.3	15	-5.5	3.72	0.08	2.08	1.99	0.84	0.74	0	283	0.12	60	0	283	1.05	1.19	0.3	0.04	1.94	3.28	1.34	0.59	0.41	0.08	0.13	0.14	0.16	0.16	0.03	0.7	1.73	0.2	0.36	0.67	0.54	1.04	0.04	6	4	0.61	9.04	0.61
161	206	MY	54	f	-4	12	-9.4	18.3	-0.2	1.92	1.31	0.88	0.73	0.01	276	0.03	61	0.01	276	1.46	1.76	0.19	0.03	1	1.95	0.94	0.51	0.49	0.02	0.11	0.34	0.27	0.2	0.04	0.19	1.09	0.33	0.19	0.61	0.94	1.25	0.25	6	4	0.5	30.1	0.5
163	231	MY	65	f	-7.8	15	-4.6	4.8	0.1	1.91	1.65	0.87	0.81	0	354	0.07	60	0.02	298	1.16	1.24	0.2	0.06	1.68	2.44	0.76	0.69	0.31	0.02	0.1	0.16	0.16	0.07	0.02	0.3	1.65	0.16	0.18	0.84	0.77	1.46	0.46	6	4	0.68	22.6	0.68
165	258	MY	47	f	-5.3	13	-0.6	5.35	0.55	2.05	1.96	0.8	0.66	0.05	292	0.11	78	0.05	292	1.05	1.26	0.23	0.19	1.55	3.08	1.54	0.5	0.5	0.12	0.12	0.14	0.21	0.19	0.07	0.35	1.14	0.25	0.22	0.4	0.64	0.72	-0.3	5	3	0.26	15.4	0.26
166	244	GE	55	m	-4.6	13	-5.1	9.09	0.09	1.83	2.09	0.89	0.81	0.01	290	0.07	74	0.01	290	0.88	0.97	0.17	0.03	2.01	2.88	0.87	0.7	0.3	0.08	0.09	0.14	0.11	0.04	0.02	0.51	1.35	0.12	0.25	0.59	0.43	0.92	-0.1	5	3	0.6	6.28	0.6
168	205	MY	63	f	-4.8	13	-9.4	12.2	-0.8	1.71	1.49	0.8	0.73	0	295	0.09	60	0	295	1.15	1.25	0.21	0.14	1.12	1.94	0.82	0.58	0.43	0.03	0.11	0.25	0.26	0.13	0.02	0.16	0.74	0.17	0.14	0.33	0.53	0.71	-0.3	6	4	0.77	16	0.77
169	235	MY	57	f	-6.3	20	1.65	2.07	0.3	1.91	1.95	0.71	0.69	0	307	0.14	60	0.02	300	0.98	1.01	0.33	0.22	1.4	2.82	1.43	0.5	0.51	0.02	0.15	0.22	0.3	0.27	0.06	0.29	1.33	0.48	0.21	0.53	1.05	1.06	0.06	7	5	0.49	20.2	0.49
169	217	SS	63	m	-4.3	17	-3.9	8.73	-0.6	1.61	1.6	0.81	0.69	0.02	351	0.1	77	0.06	298	1.01	1.18	0.17	0.14	0.99	2.04	1.05	0.49	0.51	0.06	0.1	0.18	0.3	0.3	0.08	0.09	0.57	0.15	0.09	0.38	0.37	0.69	-0.3	5	3	0.14	9.96	0.14
170	233	FI	41	m	-2.4	15	-5	3.03	0.63	1.72	1.75	0.84	0.84	0	306	0.12	119	0.01	260	0.98	0.99	0.26	0.01	1.55	2.25	0.69	0.69	0.31	0.13	0.18	0.13	0.06	0.01	0.02	0.33	0.24	0.13	0.26	0.67	0.52	0.6	-0.4	6	4	0.76	-8.7	0.76
171	221	GE	67	f	2.63	17	0.83	1.64	-0.6	1.92	1.72	0.72	0.7	0.08	296	0.17	113	0.08	296	1.12	1.17	0.35	0.18	1.29	2.61	1.33	0.49	0.51	0.14	0.18	0.18	0.17	0.14	0.09	0.31	1.16	0.27	0.24	0.52	0.43	0.95	-0	5	3	0.31	7.76	0.31
172	225	GE	36	f	0.13	13	-2.5	4.76	-0.2	2.3	1.98	0.86	0.84	0	234	0.05	114	0.01	240	1.16	1.18	0.15	0.17	2.69	3.55	0.86	0.76	0.24	0.06	0.06	0.06	0.08	0.04	0.08	0.9	1.44	0.04	0.33	0.7	0.21	0.87	-0.1	6	4	-0.2	-3.6	0.2
176	228	MY	45	m	-3.1	17	-0.7	1.71	0.21	2.17	2.15	0.84	0.81	0.01	316	0.08	60	0.02	268	1.01	1.04	0.28	0.07	2.25	3.51	1.26	0.64	0.36	0.03	0.1	0.18	0.17	0.14	0.03	0.52	1.53	0.21	0.23	0.51	0.48	0.87	-0.1	5	3	0.61	7.16	0.61
180	228	MY	52	f	-4.3	14	-6.8	9.32	-0.1	2.06	1.42	0.74	0.84	0.02	6	0.14	60	0.02	290	1.45	1.27	0.39	0.16	1.36	2.26	0.9	0.6	0.4	0.04	0.16	0.21	0.15	0.11	0.04	0.46	1.59	0.35	0.34	1.17	1.21	1.84	0.84	5	3	0.64	29.8	0.64
180	242	FI	54	m	-2.5	12	-3.4	8.57	0.24	1.63	1.63	0.67	0.63	0.09	255	0.2	60	0.09	255	1	1.08	0.36	0.18	0.83	2.07	1.24	0.4	0.6	0.17	0.21	0.23	0.21	0.13	0.15	0.12	0.84	0.16	0.15	0.44	0.39	0.87	-0.1	5	3	0.16	6.88	0.16
193	259	GE	65	m	-0.4	13	-4.7	11.8	0.49	1.92	1.76	0.88	0.88	0.01	284	0.08	117	0.01	284	1.09	1.09	0.2	0.04	2.01	2.65	0.63	0.76	0.24	0.06	0.11	0.09	0.07	0.05	0.02	0.71	1.44	0.04	0.35	0.75	0.72	1.03	0.03	6	4	0.72	5.47	0.72
