# Supplementary material for: Comparisons between retinal vessel calibers and various optic disc morphologic parameters with different optic disc appearances: The Glaucoma Stereo Analysis Study
Source: PLoS One. 2021 Jul 29;16(7):e0250245. doi: 10.1371/journal.pone.0250245 (PMC8320981; doi:10.1371/journal.pone.0250245)
Supplement: S2 File — (PDF) [file pone.0250245.s002.pdf]

S2 File. Comparison of optic nerve disc parameters among different optic disc appearances.

	Total		FI	GE	MY	SS	*p* value†
N (%)	240		53 (22)	53 (22)	112 (47)	22 (9)	
Vertical disc width, mm	1.84 ± 0.20		1.73 ± 0.16	1.91 ± 0.19	1.87 ± 0.23	1.81 ± 0.20	<0.0001
		p value‡, versus GE	<0.0001**	-	-	-	
		Versus MY	0.0009**	0.5348	-	-	
		Versus SS	0.4991	0.1874	0.6127	-	
Horizontal disc width, mm	1.66± 0.23		1.65 ± 0.17	1.84 ± 0.20	1.55 ± 0.26	1.78 ± 0.20	<0.0001
		p value‡, versus GE	<0.0001**	-	-	-	
		Versus MY	0.0502	<0.0001**	-	-	
		Versus SS	0.1109	0.6864	0.0001**	-	
Vertical cup-disc ratio	0.83 ± 0.07		0.80 ± 0.06	0.87 ± 0.070	0.82 ± 0.08	0.86 ± 0.06	<0.0001
		p value‡, versus GE	<0.0001**	-	-	-	
		Versus MY	0.3520	0.0004**	-	-	
		Versus SS	0.0071*	0.9561	0.0005**	-	
Horizontal cup-disc ratio	0.75 ± 0.08		0.72 ± 0.07	0.81 ± 0.06	0.74 ± 0.09	0.75 ± 0.09	<0.0001
		p value‡, versus GE	<0.0001**	-	-	-	
		Versus MY	0.6261	<0.0001**	-	-	

		Versus SS	0.4038	0.0178	0.8369	-	
Minimum rim-disc ratio	0.02 ± 0.02		0.02 ± 0.02	0.02 ± 0.03	0.01 ± 0.02	0.01 ± 0.02	0.5900
Minimum rim-disc ratio angle, degree	177.21 ± 122.88		227.47 ± 105.04	209.04 ± 106.10	146.41 ± 135.92	136.27 ± 129.80	<0.0001
		p value‡, versus GE	0.8669	-	-	-	
		Versus MY	0.0006**	0.0132	-	-	
		Versus SS	0.0196	0.0931	0.9848	-	
Superior minimum rim-disc ratio	0.08 ± 0.06		0.12 ± 0.06	0.07 ± 0.05	0.07 ± 0.06	0.06 ± 0.05	<0.0001
		p value‡, versus GE	<0.0001**	-	-	-	
		Versus MY	<0.0001**	0.9990	-	-	
		Versus SS	<0.0001**	0.9627	0.9238	-	
Superior minimum rim-disc ratio angle, degree	71.49 ± 16.88		77.57 ± 21.81	77.66 ± 21.17	65.80 ± 10.58	70.95 ± 18.01	<0.0001
		p value‡, versus GE	1.0000	-	-	-	
		Versus MY	0.0002**	0.0002**	-	-	
		Versus SS	0.4130	0.4001	0.5586	-	
Inferior minimum rim-disc ratio	0.03 ± 0.05		0.04 ± 0.06	0.03 ± 0.04	0.03 ± 0.04	0.03 ± 0.04	0.5951
Inferior minimum rim disc ratio angle, degree	284.79 ± 13.91		283.06 ± 14.20	277.26 ± 18.54	289.41 ± 10.27	283.59 ± 16.31	<0.0001
		p value‡, versus GE	0.1424	-	-	-	

		Versus MY	0.0332	<0.0001**	-	-	
		Versus SS	0.9988	0.2790	0.2786	-	
Disc aspect ratio	1.13 ± 0.13		1.06 ± 0.08	1.04 ± 0.08	1.23 ± 0.17	1.02 ± 0.09	<0.0001
		p value‡, versus GE	0.9573	-	-	-	
		Versus MY	<0.0001**	<0.0001**	-	-	
		Versus SS	0.7201	0.9114	<0.0001**	-	
Cup aspect ratio	1.26 ± 0.20		1.19 ± 0.16	1.13 ± 0.15	1.38 ± 0.24	1.18 ± 0.16	<0.0001
		p value‡, versus GE	0.4104	-	-	-	
		Versus MY	<0.0001**	<0.0001**	-	-	
		Versus SS	1.0000	0.6640	0.0002**	-	
Superior rim width, mm	0.19 ± 0.09		0.24 ± 0.09	0.16 ± 0.09	0.19 ± 0.10	0.16 ± 0.09	<0.0001
		p value‡, versus GE	0.0002**	-	-	-	
		Versus MY	0.0126	0.2217	-	-	
		Versus SS	0.0037**	0.9985	0.4096	-	
Inferior rim width, mm	0.11 ± 0.09		0.10 ± 0.12	0.08 ± 0.07	0.13 ± 0.09	0.09 ± 0.08	0.0031
Cup area, mm^2	1.47 ± 0.46		1.26 ± 0.33	1.93 ± 0.49	1.31 ± 0.49	1.62 ± 0.50	<0.0001
		p value‡, versus GE	<0.0001**	-	-	-	
		Versus MY	0.9141	<0.0001**	-	-	
		Versus SS	0.0141	0.0401	0.0256	-	

Disc area, mm^2	2.40 ± 0.54		2.26 ± 0.43	2.79 ± 0.54	2.25 ± 0.58	2.55 ± 0.52	<0.0001
		p value‡, versus GE	<0.0001**	-	-	-	
		Versus MY	0.9998	<0.0001**	-	-	
		Versus SS	0.1319	0.3009	0.0742	-	
Rim area, mm^2	0.93 ± 0.26		0.99 ± 0.27	0.86 ± 0.24	0.93 ± 0.25	0.93 ± 0.33	0.0759
Cup-disc area ratio	0.60 ± 0.10		0.56 ± 0.09	0.69 ± 0.08	0.57 ± 0.11	0.63 ± 0.12	<0.0001
		p value‡, versus GE	<0.0001**	-	-	-	
		Versus MY	0.8479	<0.0001**	-	-	
		Versus SS	0.0282	0.0953	0.0687	-	
Rim-disc area ratio	0.40 ± 0.10		0.44 ± 0.09	0.31 ± 0.08	0.43 ± 0.11	0.37 ± 0.12	<0.0001
		p value‡, versus GE	0.0004**	-	-	-	
		Versus MY	<0.0001**	0.0008**	-	-	
		Versus SS	<0.0001**	0.2530	0.8718	-	
Rim-disc ratio of section 1	0.06 ± 0.04		0.10 ± 0.04	0.07 ± 0.04	0.05 ± 0.04	0.05 ± 0.03	<0.0001
		p value‡, versus GE	0.0004**	-	-	-	
		Versus MY	<0.0001**	0.0008**	-	-	
		Versus SS	<0.0001**	0.2530	0.8718	-	
Rim-disc ratio of section 2	0.10 ± 0.05		0.13 ± 0.06	0.08 ± 0.04	0.09 ± 0.05	0.08 ± 0.05	<0.0001
		p value‡, versus GE	<0.0001**	-	-	-	
		Versus MY	<0.0001**	0.5608	-	-	

		Versus SS	<0.0001**	0.9729	0.4990	-	
Rim-disc ratio of section 3	0.17 ± 0.05		0.17 ± 0.05	0.11 ± 0.04	0.19 ± 0.06	0.15 ± 0.06	<0.0001
		p value‡, versus GE	<0.0001**	-	-	-	
		Versus MY	0.0336	<0.0001**	-	-	
		Versus SS	0.4078	0.0244	0.0019*	-	
Rim-disc ratio of section 4	0.19 ± 0.06		0.19 ± 0.06	0.12 ± 0.05	0.23 ± 0.07	0.20 ± 0.09	<0.0001
		p value‡, versus GE	<0.0001**	-	-	-	
		Versus MY	0.0011**	<0.0001**	-	-	
		Versus SS	0.9245	<0.0001**	0.1783	-	
Rim-disc ratio of section 5	0.14 ± 0.07		0.13 ± 0.07	0.08 ± 0.05	0.18 ± 0.08	0.13 ± 0.08	<0.0001
		p value‡, versus GE	0.0086	-	-	-	
		Versus MY	0.0001**	<0.0001**	-	-	
		Versus SS	0.9982	0.0462	0.0189	-	
Rim-disc ratio of section 6	0.06 ± 0.05		0.06 ± 0.06	0.05 ± 0.04	0.06 ± 0.05	0.05 ± 0.04	0.4824
Cup volume, mm^3	0.31 ± 0.17		0.26 ± 0.12	0.52 ± 0.19	0.25 ± 0.18	0.26 ± 0.15	<0.0001
		p value‡, versus GE	<0.0001**	-	-	-	
		Versus MY	0.9994	<0.0001**	-	-	
		Versus SS	1.0000	<0.0001**	0.9999	-	

Disc volume, mm^3	0.94 ± 0.42		0.79 ± 0.34	1.14 ± 0.44	0.96 ± 0.47	0.68 ± 0.20	<0.0001
		p value‡, versus GE	0.0002**	-	-	-	
		Versus MY	0.0760	0.0538	-	-	
		Versus SS	0.7331	0.0001**	0.0245	-	
Rim volume, mm^3	0.16 ± 0.09		0.14 ± 0.07	0.10 ± 0.06	0.20 ± 0.10	0.10 ± 0.09	<0.0001
		p value‡, versus GE	0.0382	-	-	-	
		versus MY	0.0001**	<0.0001**	-	-	
		Versus SS	0.2222	0.9990	<0.0001**	-	
Mean cup depth, mm	0.20 ± 0.06		0.20 ± 0.05	0.27 ± 0.06	0.18 ± 0.07	0.15 ± 0.06	<0.0001
		p value‡, versus GE	<0.0001**	-	-	-	
		Versus MY	0.2954	<0.0001**	-	-	
		Versus SS	0.0146	<0.0001**	0.1792	-	
Maximum cup depth, mm	0.53 ± 0.19		0.51 ± 0.13	0.65 ± 0.16	0.50 ± 0.22	0.43 ± 0.14	<0.0001
		p value‡, versus GE	0.0008**	-	-	-	
		Versus MY	0.9839	<0.0001**	-	-	
		Versus SS	0.3189	<0.0001**	0.3772	-	
Height variation contour, mm	0.55 ± 0.19		0.45 ± 0.17	0.44 ± 0.15	0.67 ± 0.22	0.42 ± 0.16	<0.0001
		p value‡, versus GE	0.9952	-	-	-	
		Versus MY	<0.0001**	<0.0001**	-	-	
		Versus SS	0.9100	0.9628	<0.0001**	-	

Depth map maximum, mm	0.87 ± 0.23		0.81 ± 0.19	0.91 ± 0.19	0.91 ± 0.27	0.64 ± 0.13	<0.0001
		p value‡, versus GE	0.0864	-	-	-	
		Versus MY	0.0328	1.0000	-	-	
		Versus SS	0.0270	<0.0001**	<0.0001**	-	
Depth map minimum, mm	-0.13 ±0.23		-0.19 ± 0.19	-0.09 ± 0.19	-0.09 ± 0.27	-0.36 ± 0.13	<0.0001
		p value‡, versus GE	0.0864	-	-	-	
		Versus MY	0.0328	1.0000	-	-	
		Versus SS	0.0270	<0.0001**	<0.0001**	-	
Rim category	5.92 ± 0.97		5.72 ± 0.74	5.87 ± 1.04	6.07 ± 1.05	5.77 ± 0.81	0.1298
DDLS stage	3.92 ± 0.97		3.72 ± 0.74	3.87 ± 1.04	4.07 ± 1.05	3.77 ± 0.81	0.1298
Rim decentering	0.28 ± 0.45		0.41 ± 0.52	0.27 ± 0.41	0.24 ± 0.45	0.20 ± 0.40	0.1327
Disc tilt angle, degree	10.39 ± 8.82		5.78 ± 6.70	2.91 ± 7.51	17.38 ± 10.17	3.95 ± 8.79	<0.0001
		p value‡, versus GE	0.3398	-	-	-	
		Versus MY	<0.0001**	<0.0001**	-	-	
		Versus SS	0.8464	0.9666	<0.0001**	-	
Rim decentration (absolute value)	0.46 ± 0.27		0.62 ± 0.24	0.40 ± 0.27	0.43 ± 0.28	0.35 ± 0.27	<0.0001
		p value‡, versus GE	0.0003**	-	-	-	
		Versus MY	0.0002**	0.9283	-	-	

versus SS	0.0008**	0.8896	0.6155	-

The *p* values were calculated among the four types of optic disc appearances by one-way analysis of variance (†) followed by comparison between each pair of two types of optic disc appearances using the post-hoc Student t-test (‡). In the post-hoc test, based on Bonferroni's method to correct multiple comparisons, $p<0.0083$ and $p<0.0017$ were considered to be significance levels of 5% (*) and 1% (**), respectively. The data are expressed as the mean ± standard error. FI, focal ischemic; GE, generalized enlargement; MY, myopic glaucomatous; SS, senile sclerotic; DDLS, disc damage likelihood scale.
