# Supplementary figures and images for: Comparisons between retinal vessel calibers and various optic disc morphologic parameters with different optic disc appearances: The Glaucoma Stereo Analysis Study
Source: PLoS One. 2021 Jul 29;16(7):e0250245. doi: 10.1371/journal.pone.0250245 (PMC8320981; doi:10.1371/journal.pone.0250245)

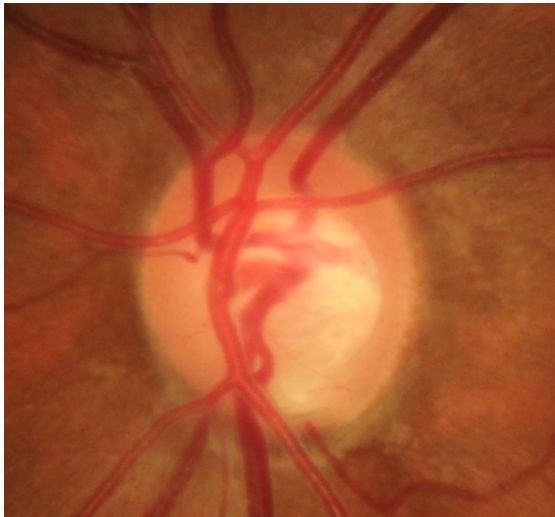

FI

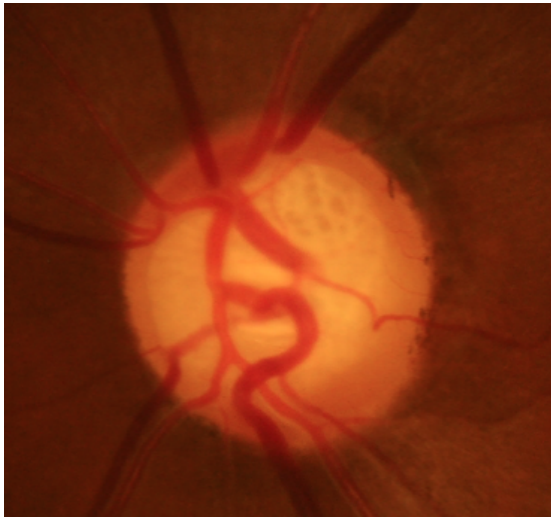

GE

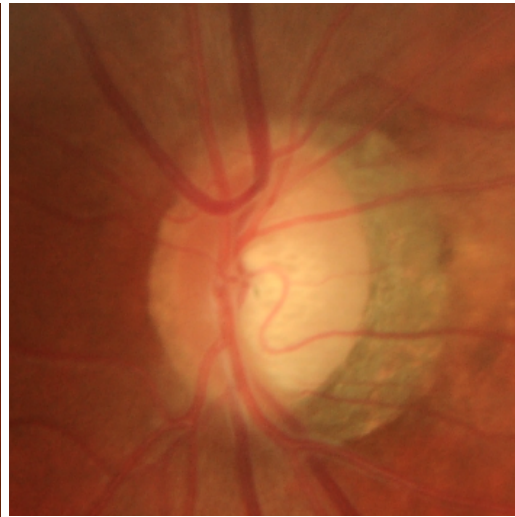

MY

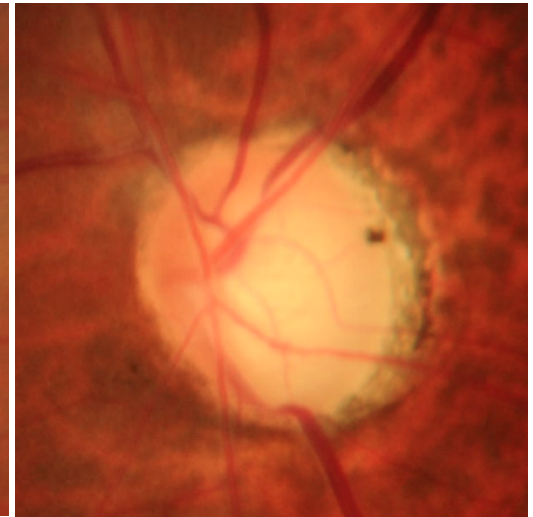

SS

S3 File. Examples of each optic disc appearance.

Supplement: S3 File — (PDF) [file pone.0250245.s003.pdf]

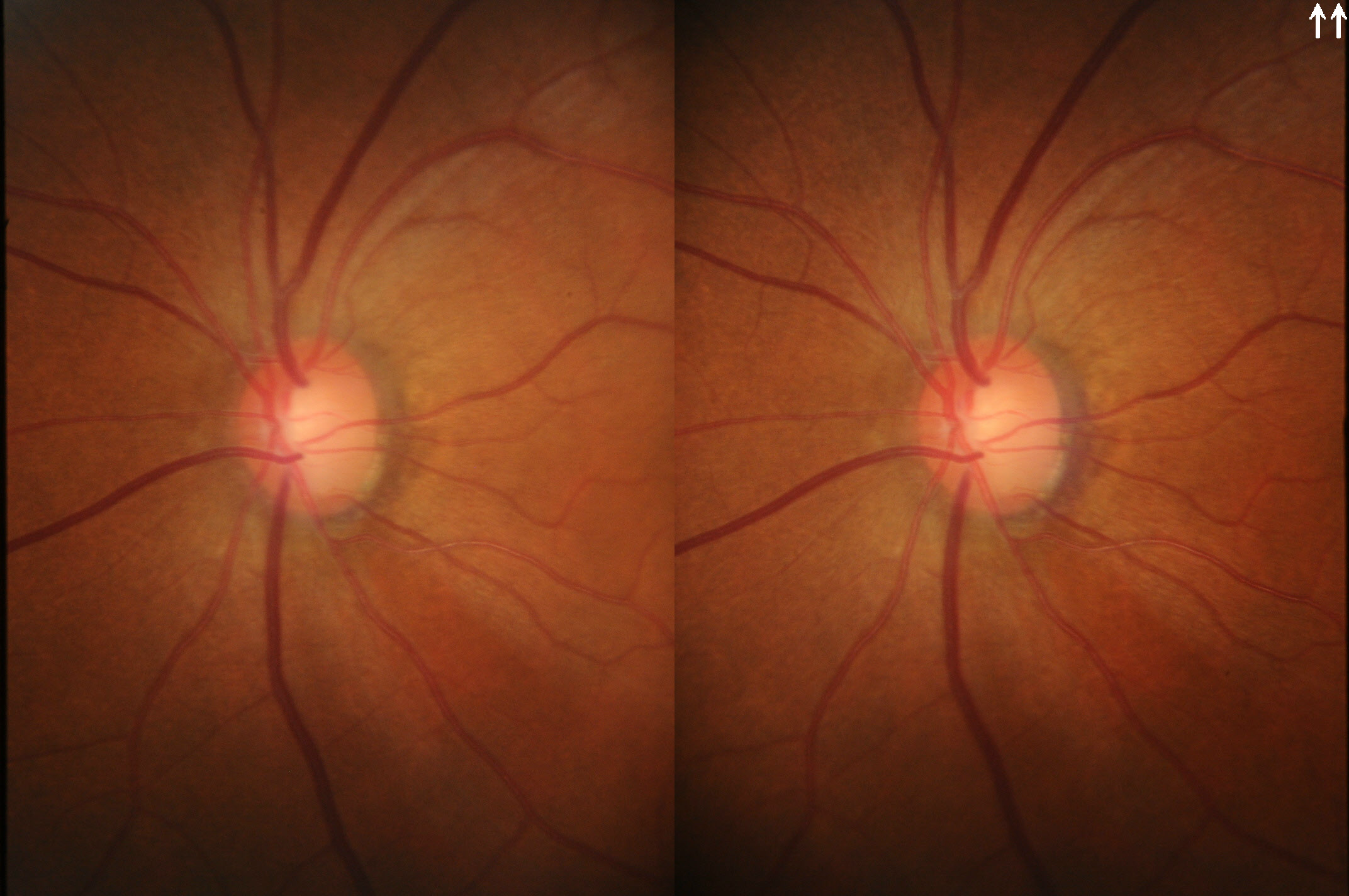

Supplement: S4 File — (JPG) [file pone.0250245.s004.jpg]

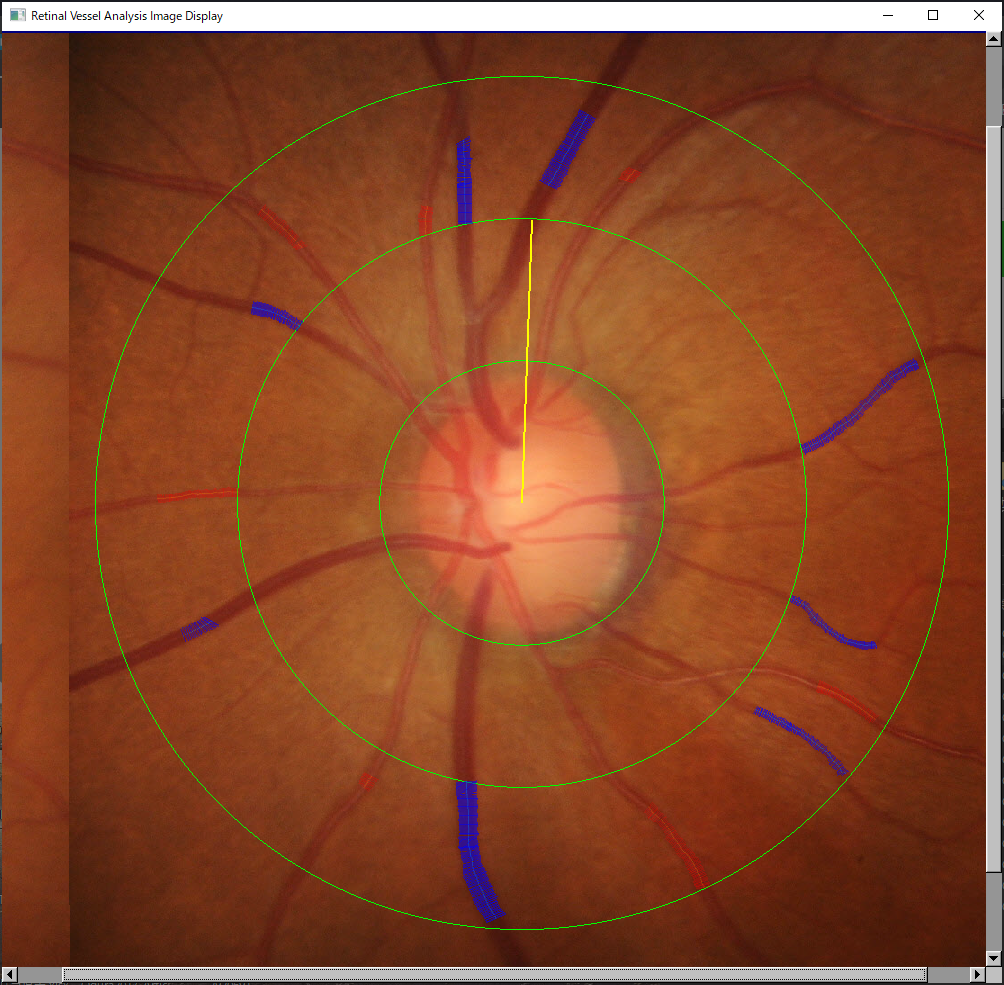

Supplement: S5 File — (PNG) [file pone.0250245.s005.png]

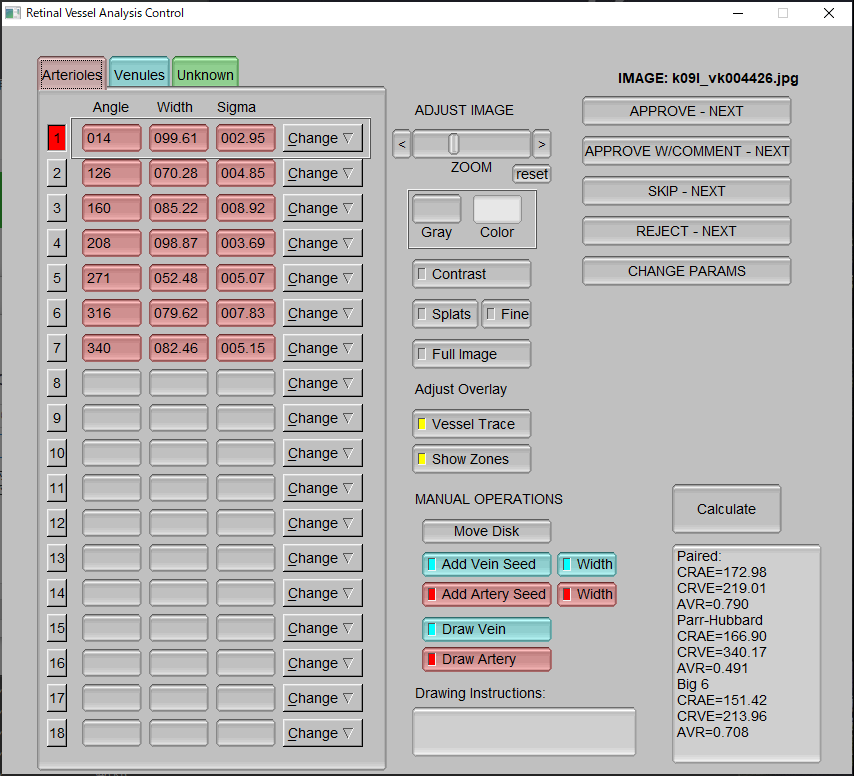

Supplement: S6 File — (PNG) [file pone.0250245.s006.png]

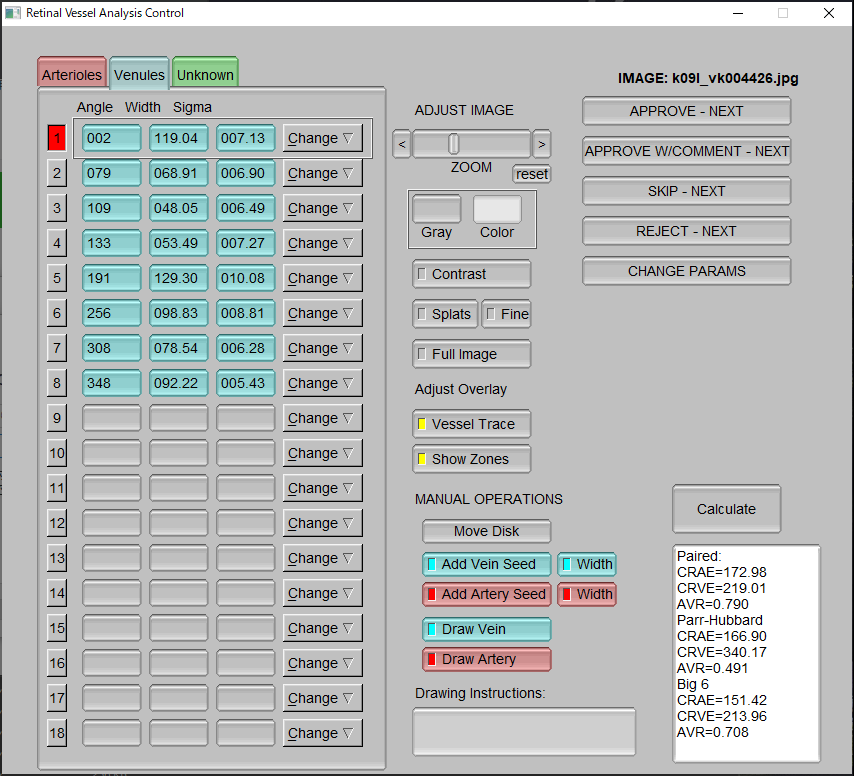

Supplement: S7 File — (PNG) [file pone.0250245.s007.png]

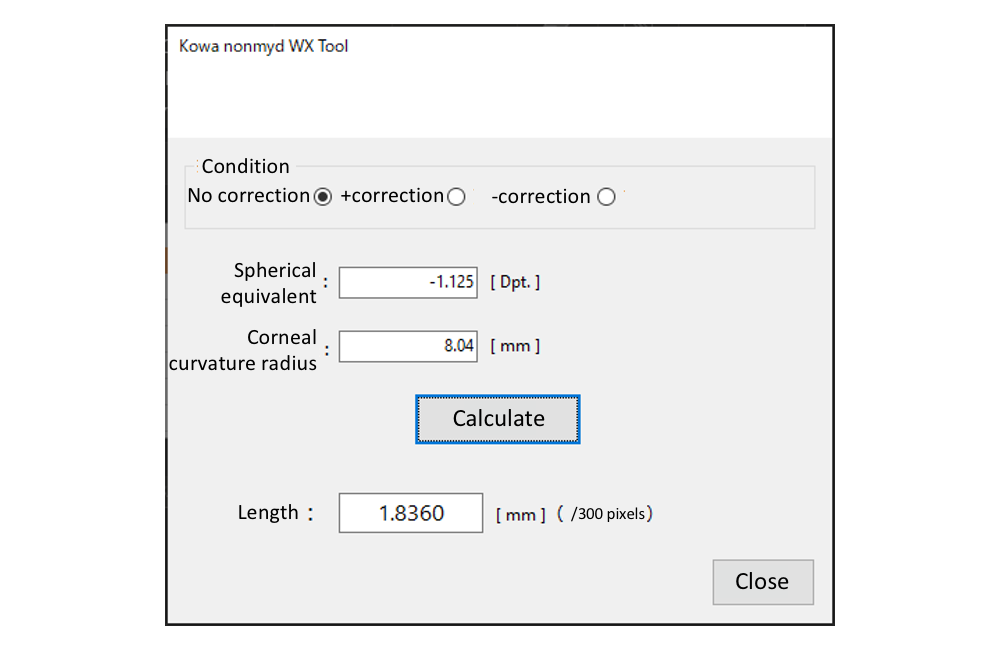

Supplement: S8 File — (PNG) [file pone.0250245.s008.png]

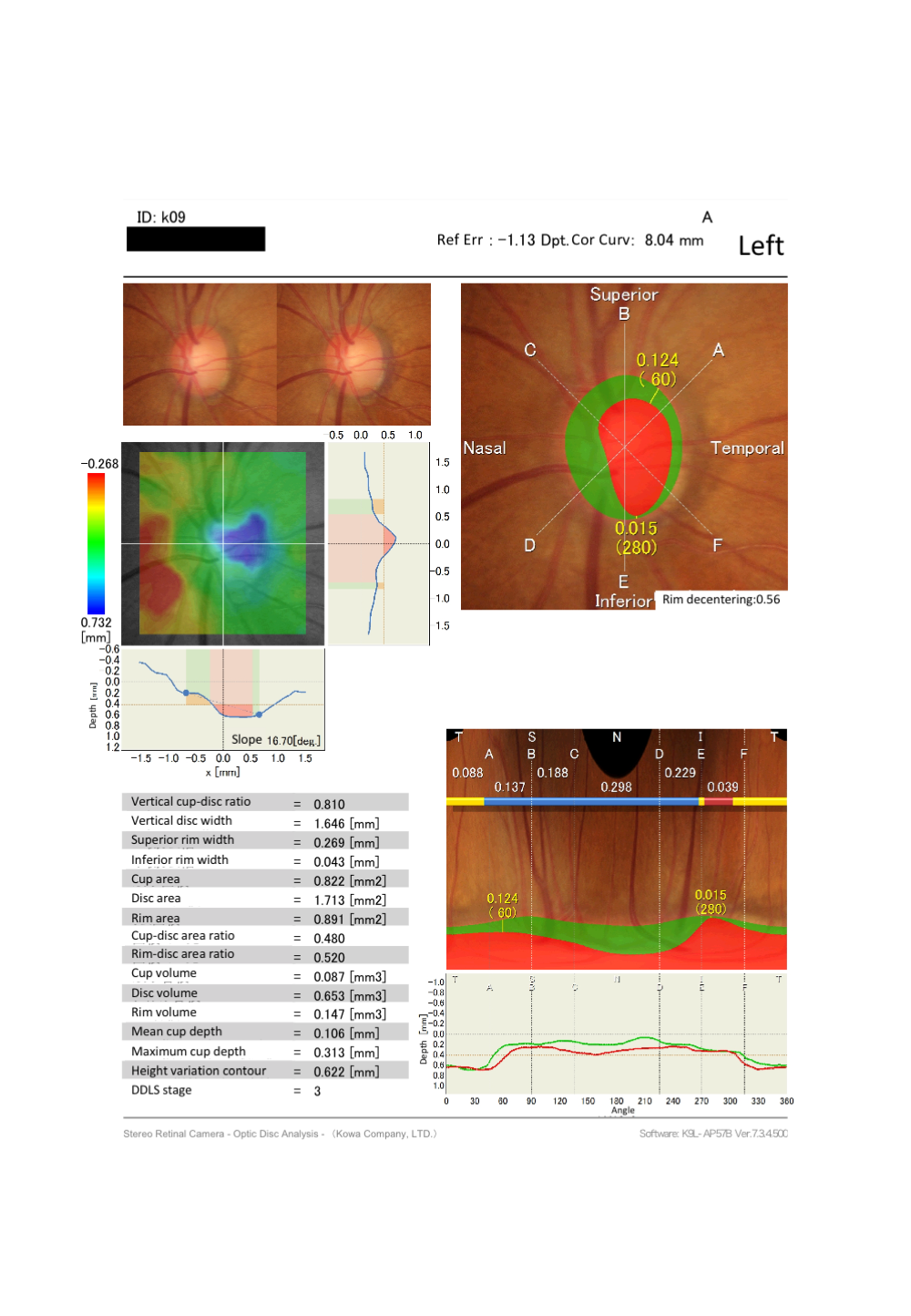

Supplement: S9 File — (PNG) [file pone.0250245.s009.png]
