# Supplementary material for: Comparisons between retinal vessel calibers and various optic disc morphologic parameters with different optic disc appearances: The Glaucoma Stereo Analysis Study
Source: PLoS One. 2021 Jul 29;16(7):e0250245. doi: 10.1371/journal.pone.0250245 (PMC8320981; doi:10.1371/journal.pone.0250245)
Supplement: S10 File — (PDF) [file pone.0250245.s010.pdf]

S10 File. Correlation between CRAE and other parameters in each optic disc appearance group

	Total		FI		GE		MY		SS	
	ρ	*p* value	ρ	*p* value	ρ	*p* value	ρ	*p* value	ρ	*p* value
CRVE	0.43	<0.0001**	0.24	0.0775	0.41	0.0022**	0.46	<0.0001**	0.61	0.0028**
Age	-0.15	0.0199*	-0.18	0.2014	-0.20	0.1560	-0.10	0.2745	-0.11	0.6137
IOP	0.10	0.1120	0.24	0.0755	0.13	0.3443	0.03	0.7832	0.14	0.5372
MD	0.21	0.0013**	0.06	0.6885	0.31	0.0239*	0.16	0.0869	0.34	0.1184
MD slope	0.19	0.0030**	0.26	0.6430	0.22	0.1221	0.17	0.0668	0.07	0.7543
PSD	-0.17	0.0052**	-0.04	0.7806	-0.35	0.0102**	-0.14	0.1278	-0.17	0.4588
Vertical disc width	0.13	0.0431*	0.12	0.4024	0.16	0.2542	0.18	0.0593	0.22	0.3297
Horizontal disc width	0.10	0.1071	0.06	0.6740	0.13	0.3602	0.18	0.0563	0.22	0.3311
Vertical cup-disc ratio	-0.19	0.0027**	-0.05	0.7117	-0.30	0.0279*	-0.09	0.3459	-0.35	0.1072
Horizontal cup-disc ratio	-0.18	0.0049**	-0.22	0.1086	-0.37	0.0058**	-0.08	0.3839	-0.05	0.8087
Minimum rim-disc ratio	0.21	0.0011**	0.27	0.0519	0.33	0.0153*	0.13	0.1568	0.11	0.6362
Minimum rim-disc ratio angle	0.24	0.0002**	-0.05	0.7131	0.40	0.0034**	0.23	0.0127*	0.10	0.6560
Superior minimum rim-disc ratio	0.20	0.0023**	-0.10	0.4710	0.18	0.1929	0.24	0.0120*	0.54	0.0089**
Superior minimum rim-disc ratio angle	0.10	0.1268	-0.10	0.4862	0.05	0.7339	0.20	0.0345*	0.18	0.4170
Inferior minimum rim-disc ratio	0.15	0.0175*	0.24	0.0855	0.30	0.0302*	0.03	0.7392	-0.01	0.9741
Inferior minimum rim disc-ratio angle	0.06	0.3741	-0.01	0.9696	0.21	0.1351	-0.02	0.8162	0.08	0.7298
Disc aspect ratio	-0.04	0.5383	0.14	0.3249	0.03	0.8471	-0.15	0.1239	0.00	0.9861
Cup aspect ratio	-0.03	0.6323	0.15	0.2779	0.00	0.9991	-0.13	0.1813	-0.10	0.6434
Superior rim width	0.21	0.0010**	-0.06	0.6775	0.26	0.0651	0.20	0.0307*	0.42	0.0526
Inferior rim width	0.13	0.0518	0.15	0.2819	0.34	0.0118*	0.00	0.9709	0.04	0.8515
Cup area	-0.02	0.7518	-0.09	0.5192	-0.09	0.5000	0.09	0.3497	0.03	0.9106
Disc area	0.13	0.0384*	0.11	0.4144	0.11	0.4266	0.22	0.0209*	0.24	0.2867
Rim area	0.33	<0.0001**	0.25	0.0661	0.39	0.0037**	0.29	0.0018**	0.37	0.0935
Cup-disc area ratio	-0.21	0.0012**	-0.21	0.1279	-0.36	0.0087**	-0.13	0.1727	-0.24	0.2821

Rim-disc area ratio	0.21	0.0014**	0.21	0.1247	0.35	0.0091**	0.13	0.1879	0.24	0.2821
Rim-disc ratio of section 1	0.13	0.0382*	0.13	0.3567	0.04	0.7557	0.15	0.1085	0.14	0.5385
Rim-disc ratio of section 2	0.20	0.0023**	-0.02	0.8845	0.21	0.1234	0.20	0.0328*	0.38	0.0791
Rim-disc ratio of section 3	0.11	0.0866	0.14	0.3114	0.25	0.0730	0.03	0.7332	0.07	0.7473
Rim-disc ratio of section 4	0.10	0.1287	0.17	0.2145	0.28	0.0415*	0.03	0.7363	0.05	0.8398
Rim-disc ratio of section 5	0.11	0.0854	0.19	0.1653	0.29	0.0386*	0.00	0.9761	0.12	0.6007
Rim-disc ratio of section 6	0.15	0.0229*	0.22	0.1216	0.26	0.0631	0.03	0.7913	0.00	0.9881
Cup volume	0.01	0.9159	-0.09	0.5383	-0.03	0.8049	0.11	0.2596	0.17	0.4434
Disc volume	0.15	0.0188*	0.01	0.9662	0.24	0.0814	0.23	0.0138*	0.20	0.3656
Rim volume	0.19	0.0040**	0.09	0.5196	0.30	0.0305*	0.20	0.0345*	0.46	0.0296*
Mean cup depth	0.02	0.7369	-0.12	0.3742	0.09	0.5380	0.07	0.4721	0.24	0.2902
Maximum cup depth	0.06	0.3540	-0.05	0.7156	0.02	0.9081	0.12	0.2155	0.11	0.6115
Height variation contour	0.02	0.7712	-0.02	0.8666	0.08	0.5457	-0.02	0.8643	0.37	0.0861
Depth map maximum	0.12	0.0703	-0.08	0.5789	0.17	0.2151	0.18	0.0634	0.20	0.3653
Depth map minimum	0.12	0.0703	-0.08	0.5789	0.17	0.2151	0.18	0.0634	0.20	0.3653
Rim category	-0.25	0.0001**	-0.33	0.0144*	-0.37	0.0070**	-0.14	0.1326	-0.24	0.2738
DDLS stage	-0.25	0.0001**	-0.33	0.0144*	-0.37	0.0070**	-0.14	0.1326	-0.24	0.2738
Rim decentering	0.01	0.8434	-0.20	0.1573	-0.12	0.3966	0.13	0.1631	0.27	0.2234
Disc tilt angle	0.00	0.9702	-0.09	0.5368	-0.14	0.3006	-0.01	0.9454	0.10	0.6727
Rim decentering (absolute value)	-0.05	0.4620	-0.29	0.0378*	0.00	0.9854	0.01	0.9431	0.20	0.3822

The *p* values and correlation coefficients (ρ) between CRAE and each parameter were calculated using the Spearman's rank correlation coefficient test in each disc appearance group. * and ** indicate $p<0.05$ and $p<0.01$, respectively.

FI, focal ischemic; GE, generalized enlargement; MY, myopic glaucoma; SS, senile sclerosis; CRAE, central retinal arteriolar equivalent; CRVE, central retinal venular equivalent; SERE, spherical equivalent refractive error; MD, mean deviation; PSD, pattern standard deviation; DDLS, disc damage likelihood scale.
