# Supplementary material for: Comparisons between retinal vessel calibers and various optic disc morphologic parameters with different optic disc appearances: The Glaucoma Stereo Analysis Study
Source: PLoS One. 2021 Jul 29;16(7):e0250245. doi: 10.1371/journal.pone.0250245 (PMC8320981; doi:10.1371/journal.pone.0250245)
Supplement: S11 File — (PDF) [file pone.0250245.s011.pdf]

S11 File. Correlation between CRVE and other parameters in each optic disc appearance group

	Total		FI		GE		MY		SS	
	ρ	*p* value	ρ	*p* value	ρ	*p* value	ρ	*p* value	ρ	*p* value
CRAE	0.43	<0.0001**	0.24	0.0775	0.41	0.0022**	0.46	<0.0001**	0.61	0.0028**
Age	-0.31	<0.0001**	-0.36	0.0072**	-0.19	0.1759	-0.26	0.0054**	-0.37	0.0876
IOP	0.18	0.0050**	0.18	0.1902	0.36	0.0076**	0.21	0.0295*	-0.13	0.5671
MD	0.15	0.0164*	0.18	0.2080	0.18	0.2024	0.17	0.0783	0.21	0.3375
MD slope	0.19	0.0038**	0.22	0.1217	0.30	0.0305*	0.15	0.1192	0.11	0.6355
PSD	-0.10	0.1287	-0.15	0.2933	-0.15	0.2744	-0.17	0.0737	0.08	0.7227
Vertical disc width	0.20	0.0017**	0.12	0.4049	0.09	0.5262	0.32	0.0006**	0.42	0.0489*
Horizontal disc width	0.14	0.0273*	0.21	0.1252	-0.01	0.9349	0.35	0.0002**	0.46	0.0330*
Vertical cup-disc ratio	-0.08	0.2465	-0.17	0.2338	-0.09	0.5381	0.04	0.6961	-0.11	0.6185
Horizontal cup-disc ratio	-0.13	0.0404*	-0.30	0.0312*	-0.18	0.1904	0.03	0.7585	-0.07	0.7615
Minimum rim-disc ratio	0.04	0.4955	0.03	0.8336	0.18	0.2047	0.03	0.7627	0.03	0.8866
Minimum rim-disc ratio angel	0.08	0.2162	0.09	0.5427	0.10	0.4925	0.07	0.4854	0.03	0.9006
Superior minimum rim-disc ratio	0.06	0.3559	-0.02	0.8675	0.00	0.9906	0.07	0.4442	0.46	0.0326*
Superior minimum rim-disc ratio angle	0.08	0.2266	0.00	0.9887	0.10	0.4667	0.24	0.0104*	-0.12	0.5916
Inferior minimum rim-disc ratio	0.06	0.3352	0.12	0.4067	0.17	0.2195	0.00	0.9958	-0.20	0.3744
Inferior minimum rim disc-ratio angle	0.05	0.4066	0.11	0.4403	0.08	0.5524	0.02	0.8306	-0.24	0.2728
Disc aspect ratio	0.03	0.6450	-0.05	0.6991	0.19	0.1834	-0.17	0.0763	-0.02	0.9393
Cup aspect ratio	0.04	0.5237	0.08	0.5596	0.13	0.3699	-0.15	0.1094	0.02	0.9185
Superior rim width	0.10	0.1094	0.05	0.7012	0.03	0.8511	0.08	0.3789	0.36	0.0989
Inferior rim width	0.11	0.1031	0.23	0.1046	0.21	0.1286	0.02	0.8476	-0.22	0.3245
Cup area	0.06	0.3734	-0.05	0.7326	-0.07	0.6231	0.25	0.0069**	0.31	0.1675
Disc area	0.19	0.0030**	0.18	0.1952	0.03	0.8468	0.37	<0.0001**	0.48	0.0223*
Rim area	0.31	<0.0001**	0.37	0.0059**	0.14	0.3105	0.33	0.0004**	0.44	0.0388*

Cup-disc area ratio	-0.13	0.0467*	-0.28	0.0424*	-0.17	0.2174	0.03	0.7902	-0.09	0.6929
Rim-disc area ratio	0.13	0.0448*	0.28	0.0420*	0.18	0.2076	-0.03	0.7902	0.09	0.6929
Rim-disc ratio of section 1	0.08	0.1931	0.03	0.8162	0.11	0.4416	0.11	0.2345	0.13	0.5570
Rim-disc ratio of section 2	0.05	0.4109	-0.01	0.9215	0.05	0.7044	0.03	0.7541	0.26	0.2429
Rim-disc ratio of section 3	0.05	0.4547	0.20	0.1475	0.01	0.9358	-0.17	0.0795	0.07	0.7606
Rim-disc ratio of section 4	0.10	0.1057	0.34	0.0122*	0.13	0.3679	-0.12	0.2076	0.05	0.8281
Rim-disc ratio of section 5	0.12	0.0538	0.26	0.0616	0.18	0.1905	-0.02	0.8101	0.00	0.9940
Rim-disc ratio of section 6	0.05	0.4366	0.12	0.4029	0.19	0.1812	-0.03	0.7874	-0.27	0.2332
Cup volume	0.09	0.1544	0.08	0.5486	0.02	0.8888	0.28	0.0028*	0.18	0.4298
Disc volume	0.25	0.0001**	0.29	0.0343*	0.09	0.5224	0.30	0.0012*	0.32	0.1410
Rim volume	0.25	<0.0001**	0.31	0.0219*	0.09	0.5386	0.16	0.0896	0.49	0.0207*
Mean cup depth	0.09	0.1510	0.13	0.3444	0.05	0.7234	0.21	0.0295*	0.04	0.8495
Maximum cup depth	0.12	0.0580	0.15	0.2744	-0.17	0.2329	0.25	0.0087**	0.04	0.8711
Height variation contour	0.10	0.1069	0.21	0.1324	-0.04	0.7823	0.03	0.7652	0.28	0.2144
Depth map maximum	0.21	0.0008**	0.34	0.0116*	-0.11	0.4291	0.24	0.0114*	0.30	0.1689
Depth map minimum	0.21	0.0008**	0.34	0.0116*	-0.11	0.4291	0.24	0.0114*	0.30	0.1689
Rim category	-0.08	0.2206	-0.13	0.3612	-0.14	0.3116	-0.06	0.5456	-0.13	0.5736
DDLS stage	-0.08	0.2206	-0.13	0.3612	-0.14	0.3116	-0.06	0.5456	-0.13	0.5736
Rim decentering	-0.03	0.6211	-0.13	0.3408	-0.28	0.0449	0.00	0.9615	0.36	0.1012
Disc tilt angle	0.07	0.2571	0.05	0.7098	-0.03	0.8098	-0.10	0.2759	0.23	0.3096
Rim decentering (absolute value)	-0.04	0.5579	-0.10	0.4617	-0.29	0.0330	-0.05	0.6259	0.14	0.5375

The *p* values and correlation coefficients (ρ) between CRVE and each parameter were calculated using the Spearman's rank correlation coefficient test in each disc appearance group. * and ** indicate $p<0.05$ and $p<0.01$, respectively. DDLS, disc damage likelihood scale.

FI, focal ischemic; GE, generalized enlargement; MY, myopic glaucoma; SS, senile sclerosis; CRAE, central retinal arteriolar equivalent; CRVE, central retinal venular equivalent; SERE, spherical equivalent refractive error; MD, mean deviation; PSD, pattern standard deviation; DDLS, disc damage likelihood scale.
